# Cyclic nucleotide-gated ion channel gene family in rice, identification, characterization and experimental analysis of expression response to plant hormones, biotic and abiotic stresses

**DOI:** 10.1186/1471-2164-15-853

**Published:** 2014-10-04

**Authors:** Zarqa Nawaz, Kaleem Ullah Kakar, Mumtaz A Saand, Qing-Yao Shu

**Affiliations:** State Key Laboratory of Rice Biology, Zhejiang University, Hangzhou, 310029 China; Institute of Biotechnology, Zhejiang University, Hangzhou, China; Institute of Crop Sciences, Zhejiang University, Hangzhou, 310029 China; Department of Botany, Shah Abdul Latif University, Khairpur mir’s, Sindh Pakistan

**Keywords:** CNGCs, Rice, Biotic and abiotic stress, Bioinformatics, Phylogenetic analysis, Resistance

## Abstract

**Background:**

Cyclic nucleotide-gated channels (*CNGCs*) are Ca^2+^-permeable cation transport channels, which are present in both animal and plant systems. They have been implicated in the uptake of both essential and toxic cations, Ca^2+^ signaling, pathogen defense, and thermotolerance in plants. To date there has not been a genome-wide overview of the *CNGC* gene family in any economically important crop, including rice (*Oryza sativa* L.). There is an urgent need for a thorough genome-wide analysis and experimental verification of this gene family in rice.

**Results:**

In this study, a total of 16 full length rice *CNGC* genes distributed on chromosomes 1–6, 9 and 12, were identified by employing comprehensive bioinformatics analyses. Based on phylogeny, the family of *OsCNGCs* was classified into four major groups (I-IV) and two sub-groups (IV-A and IV- B). Likewise, the *CNGCs* from all plant lineages clustered into four groups (I-IV), where group II was conserved in all land plants. Gene duplication analysis revealed that both chromosomal segmentation (*OsCNGC1* and *2, 10* and *11*, *15* and *16*) and tandem duplications (*OsCNGC1* and *2*) significantly contributed to the expansion of this gene family. Motif composition and protein sequence analysis revealed that the CNGC specific domain “cyclic nucleotide-binding domain (CNBD)” comprises a “phosphate binding cassette” (PBC) and a “hinge” region that is highly conserved among the OsCNGCs. In addition, OsCNGC proteins also contain various other functional motifs and post-translational modification sites. We successively built a stringent motif: (LI-X(2)-[GS]-X-[FV]-X-G-[1]-ELL-X-W-X(12,22)-SA-X(2)-T-X(7)-[EQ]-AF-X-L) that recognizes the rice CNGCs specifically. Prediction of cis-acting regulatory elements in 5′ upstream sequences and expression analyses through quantitative qPCR demonstrated that *OsCNGC* genes were highly responsive to multiple stimuli including hormonal (abscisic acid, indoleacetic acid, kinetin and ethylene), biotic (*Pseudomonas fuscovaginae* and *Xanthomonas oryzae* pv. *oryzae*) and abiotic (cold) stress.

**Conclusions:**

There are 16 *CNGC* genes in rice, which were probably expanded through chromosomal segmentation and tandem duplications and comprise a PBC and a “hinge” region in the CNBD domain, featured by a stringent motif. The various cis-acting regulatory elements in the upstream sequences may be responsible for responding to multiple stimuli, including hormonal, biotic and abiotic stresses.

**Electronic supplementary material:**

The online version of this article (doi:10.1186/1471-2164-15-853) contains supplementary material, which is available to authorized users.

## Background

Calcium signal transduction through calcium channels is a fundamental mechanism used by plants to sense and respond to endogenous and environmental stimuli [1], including hormone responses [1], plant–pathogen interaction [2], development [3], symbiosis [4], salt stress [5], light signaling [6], and circadian rhythm [7]. One of the potential pathways for the uptake of Ca^2+^ ions in signal transduction is via cyclic nucleotide-gated ion channels (*CNGCs*) [8]. CNGCs, functionally defined as ligand-gated protein channels, are nonselective cation channels, gated by the direct binding of cyclic nucleotides, and are present in both animals and plants system [9–12]. In animals, their biological roles and regulation have been well studied, however, plant *CNGCs* have only been investigated much more recently, and corresponding regulatory mechanisms are just starting to be discovered [13, 14]. Studies have revealed that CNGCs may function as a pathway for Ca^2+^ conduction into the cytosol as an early event during pathogen defense signaling cascades [15], and plant response to other biotic and abiotic stresses [16].

Plant CNGC ion channels were first identified in a screen for calmodulin (CaM) binding partners in barley [17]. A large family of CNGCs composed of 20 members have been identified in *Arabidopsis* genome [8] which are classified into four groups (groups I–IV) and two sub-groups “IV-A and IV-B” [18]. These CNGCs are characterized by general structural resemblance to animal CNGCs [8], having a predicted structure of six transmembrane domains (S1–S6) with a pore domain (P loop) between S5 and S6, C-terminal Cyclic nucleotide-binding domain (CNBD) and CaM-binding domains (CaMBD) [8, 13, 19, 20]. In the presence of Ca^2+^, CaM binds to the CNGC at the αC helix of the CNBD and thus blocks channel gating by cNMPs [21]. CNBD being most conserved region of the CNGC with a phosphate binding cassette (PBC) and a “hinge” region (adjacent to PBC). Where, the PBC binds to the sugar and phosphate moieties of the cyclic nucleotide (cNMP/ Cyclic nucleotide-monophosphate) ligand [22], and the hinge region contributes to ligand binding efficacy and selectivity [23]. In *Arabidopsis*, *CNGC* genes are differentially expressed in all tissues and their functions critically depend on the presence of cAMP and/or cGMP that function as signaling molecules [24–26]. Physiological processes in which these signaling molecules are believed to be involved include various developmental processes, photo-morphogenesis and tolerance to salt stress [27, 28], gibberellic acid-induced signaling in barley [29] and phytochrome signaling [30].

Approximately 5% of the *Oryza sativa* genome appears to encode membrane transport proteins. Many of these proteins are classified and several hundred putative transporters have not yet been assigned to families, including the CNGC gene family [18, 26]. The early investigations on rice genome, revealed a clue about the existence of putative *CNGC* fragments, as suggested by Bridges et al. [31] and Ramanjaneyulu et al. [29]. However, no comprehensive study has yet been undertaken, which can lead to the proper identification, characterization and functional verification of the *CNGC* family in rice.

Taking privilege from the accessibility of recent release of entire genome sequences of rice and information available from *A. thaliana*, we performed a genome-wide identification of *CNGC* family in rice in the present study. We found that the rice genome contains 16 *CNGC* full length genes which are classified into four major classes and two sub-groups as in *Arabidopsis*. Evolutionary history reconstruction via comparison with *CNGCs* from other plant species was accomplished. Furthermore, comprehensive analyses were performed to characterize these genes and protein sequence features e.g., domain, motif, phylogenetic relationships, gene structure, multiple alignment, cis-acting regulatory elements etc. To verify their functions, we analyzed the expression pattern of rice CNGC genes by quantitative PCR (qPCR) in response to various hormonal, biotic and abiotic stresses. The present work represents the first comprehensive study of CNGC genes in rice and offers a solid base for further functional investigation of this important gene family in rice.

## Results and discussion

### Identification of CNGC genes in rice genome

To obtain a complete overview of CNGC gene family in rice, we conducted a genome-wide analysis by using various bioinformatics resources. Considering the fact that the *Arabidopsis* CNGC gene family is fully characterized, we used the amino acid (aa) sequences of 20 *Arabidopsis* CNGC genes as the queries in BLAST searches and the keyword ‘cyclic nucleotide-gated ion channel’ in the putative function search tool of rice database (RAP). Seventeen non-redundant putative gene sequences were retrieved in rice with five genes (*LOC_Os02g15580, LOC_Os09g38580, LOC_Os06g08850, LOC_Os02g53340* and *LOC_Os06g10580*) having potential alternative splice forms.

Since plant CNGCs, are characterized by the presence of CNBD, specifically phosphate binding cassettes and hinge region [8, 11, 32, 33], thus their presence in a protein sequence is a validation criteria for these genes. The sequence searches of databases based on annotation are not completely accurate and could potentially miss some bonafide CNGC sequences or inaccurately assign certain sequences as CNGCs [34]. Therefore, the deduced protein sequences of all the 17 putative genes were further analyzed to confirm the presence of CNBD/Cyclic Nucleotide-Monophosphate Binding Domain (cNMP, cNMP_bd or cNMP_bd like), Cap Family Effector Domain (CAP_ED), RmlC-like jelly roll fold (RmlC), and ion transport domains. We found that *LOC*_*Os06g33600*, previously designated as “genomic cyclic nucleotide-gated ion channel 1”, was identified as “Cyclic nucleotide-gated channel C (Fragment)”, and was eradicated from analysis due to lack of above cited domains. However, a 217 aa short fragment, *LOC_Os06g33610* (latterly designated as CNGC3) contained the CNGC characteristic CNBD domain, with phosphate binding cassettes and hinge region as suggested by Zelman et al. [11], was recognized as bonafide CNGC. These identifications were purely based on the presence of primary CNBD domain, and the validation of the consensus motif derived by Zelman et al. [11]. We noticed that blast search of *Arabidopsis* CNGC also resulted into gene from potassium AKT /KAT channels (Shaker type) as homologs of CNGCs, due to the presence of transmembrane region, CNBD and ion transport domains and additional ankyrin repeats [35]. Such unrelated genes were rejected from analysis. Finally, 16 full length *CNGC* genes were confirmed to contain the specific domains in their proteins sequences, and hence were identified as *CNGC* genes in rice (Table 1).

**Table 1:** **Domain architecture of** ***OsCNGC*** **gene family**

Group	Gene Symbol	Gene Locus (Loc_)	RAP-DB ID	Domain architectures in different databases ^1^					
				Pfam	SMART	CDD	PROSITE	SUPERFAMILY	GENE3D
**Group I**	*OsCNGC*1	Os02g15580	Os02g0255000	[1] & [2]	[4]	[5] & [6]	[9]	[10]	[11]
	*OsCNGC*2	Os06g33570	Os06g0527100	[1] & [2]	[4]	[5] & [1] & [6]	[9]	[10]	[11]
	*OsCNGC*3	Os06g33610	-	[2]	[4]	[5]	[9]	[10]	[11]
**Group II**	*OsCNGC*4	Os03g44440	Os03g0646300	[1] & [2]	[4]	[5] & [1] & [6]	[9]	[10]	[11]
	*OsCNGC*5	Os12g28260	Os12g0468500	[1] & [2]	[4]	[5] & [6]	[9]	[10]	[11]
	*OsCNGC*6	Os04g55080	Os04g0643600	[1] & [2]	[4]	[5] & [1] & [6]	[9]	[10]	[11]
**Group III**	*OsCNGC*7	Os02g41710	Os02g0627700	[1] & [2]	[4]	[5] & [6]	[9]	[10]	[11]
	*OsCNGC*8	Os12g06570	Os12g0163000	[1] & [2]	[4]	[5] & [1] & [6]	[9]	[10]	[11]
	*OsCNGC*9	Os09g38580	Os09g0558300	[1] & [2]	[4]	[5] & [7]& [8]	[9]	[10]	[11]
	*OsCNGC*10	Os02g54760	Os02g0789100	[1] & [2]	[4]	[5] & [1] & [6]	[9]	[10] & [8]	[11]
	*OsCNGC*11	Os06g08850	Os06g0188000	[1] & [2]	[4]	[5] & [1] & [6]	[9]	[10]	[11]
**Group Iva**	*OsCNGC*12	Os02g53340	Os02g0773400	[3]	[4]	[5]	[9]	[10]	[11]
	*OsCNGC*13	Os06g10580	Os06g0207700	[3]	[4]	[5]	[9]	[10] & [8]	[11]
**Group IVb**	*OsCNGC*14	Os03g55100	Os03g0758300	[1] & [2]	[4]	[5]	[9]	[10] & [8]	[11]
	*OsCNGC*15	Os01g57370	Os01g0782700	[1] & [2]	[4]	[5] & [6]	[9]	[10]	[11]
	*OsCNGC*16	Os05g42250	Os05g0502000	[1] & [2]	[4]	[5] & [6]	[9]	[10]	[11]

^1^[1]: ITP (Ion transport domain), [2] cNMP_bd (Cyclic nucleotide- monophosphate binding domain or Cyclic nucleotide-binding domain), [3] Is- cNMP_bd (Insignificant Cyclic nucleotide- monophosphate binding domain). [4] Cyclic nucleotide- monophosphate, [5] CAP_ED (Cap family effector domain, which binds cAMP), TM (Transmembrane), [6] Crp (cAMP-binding proteins); [7] CCER1 (Coiled-coil domain-containing glutamate-rich protein family 1), [8] PLN (Voltage-dependent potassium channel; Provisional), [9] cNMP_bd (Cyclic nucleotide-binding domain), [10] cNMP_bd-like (Cyclic nucleotide-binding-like domain), [11] RmlC (RmlC-like jelly roll fold).

### OsCNGCs chromosomal localization and diversification

**Figure 1:**
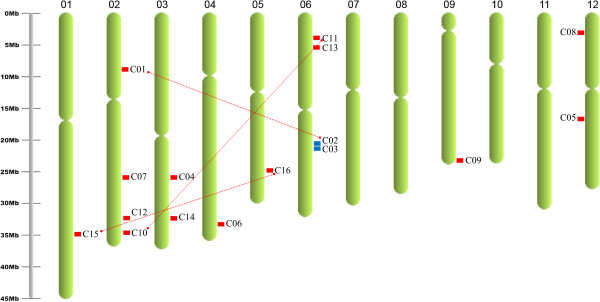
**Genomic localization and duplication of CNGC genes in rice chromosomes.** Chromosome numbers are indicated at the top of each chromosome. Genes are marked in Abbreviations i.e. C stands for *CNGC*; those present on duplicated segments of genome are connected by red lines and tandem duplicated genes are marked in blue.

Chromosome localization analysis demonstrated that the *OsCNGCs* are located on eight rice chromosomes (1, 2, 3, 4, 5, 6, 9 and 12) (Figure 1). To better reflect the orthologous relationship between the rice and *Arabidopsis CNGC* genes, the nomenclature of respective rice *CNGC* genes was annotated according to their order in phylogenies, with *Os* abbreviating *O. sativa* (Table 1).

*Arabidopsis* has been reported to have 20 *CNGC* genes (Mäser et al. 2001) [18]. Conversely, in rice that has a genome size nearly three times as that of *Arabidopsis* (Project IRGS 2005, Vij et al. 2006) [36, 37], the number of *CNGC* genes was found to be only 16. The reason for this could be the variable status of whole genome duplications in *Arabidopsis* and rice (Paterson et al. 2004, Yu et al. 2005) [38, 39]. In this study, both segmental and tandem duplication events were investigated for elucidating the potential mechanism of evolution of *OsCNGC* gene family in rice. Analysis of PGDD revealed that three genes pairs, *OsCNGC1* and *OsCNGC2*, *OsCNGC10* and *OsCNGC11*, as well as *OsCNGC15* and *OsCNGC16* located on different chromosomes regions, might have been induced by segmental duplication, and could be assigned to RGAP segmental duplication blocks (Figure 1). In addition, one gene pair, *OsCNGC*2 and *OsCNGC*3 were located at the adjacent positions on chromosome 2 and separated by 3 genes only. Hence suggested, that these two genes were generated from tandem duplication event (Figure 1). These results indicate that gene duplication not only expanded the number of genes in the rice CNGC family but also increased its functional diversity (see Gene expression analysis).

### Gene structure and features of OsCNGC proteins

Comparisons between genomic DNA sequences with corresponding cDNA sequences showed that coding sequences of *OsCNGC* genes are interrupted by introns. Introns may interrupt the reading frame of a gene between two consecutive codons (phase 0 introns), between the first and second nucleotide of a codon (phase 1 introns), or between the second and the third nucleotide (phase 2 introns) [40]. Gene structural analysis showed that the rice *CNGCs* had different gene structures as compared to *Arabidopsis CNGCs*, with a variable number of introns and lengths. The numbers of introns in *Arabidopsis CNGCs* ranged from 4 to 10 introns, while rice *CNGC* ranged from 1 to 11 introns. Unlike the *Arabidopsis CNGCs*, introns of all *OsCNGCs* belong to phase 0 and 2 (Additional file 1; Additional file 2), while, *Arabidopsis CNGC6* contained, four phase 1 introns. This suggests that all *CNGCs* with introns did not ascend from a retrotransposition event, and the different numbers may be caused by the insertion and loss of introns during their evolution [41].

The 16 OsCNGC proteins range in length from 217 (OsCNGC3) to 772 (OsCNGC12) amino acids (aa), with an average of 661 aa. ExPASy analysis showed that OsCNGC proteins vary greatly in isoelectric point (pI) values (ranging from 5.81 to 9.71) and molecular weights (ranging from 25.4 to 88,353 kDa). According to their instability index values, only 1 (OsCNGC8) of 16 OsCNGC proteins can be considered as stable with an instability index of < = 40 (Table 2) [42].

**Table 2:** **Predicted sequence features of OsCNGC proteins**

Protein ID	Length	MW.	pI	Subcellular localization	Stability	cAMP- cGMP	Casein	PKC	Tyr	N-Myr	N-Glyc
**OsCNGC1**	498	57105.7	8.88	P.m	unstable	1	5	6	-	10	5
**OsCNGC2**	694	80194.2	9.28	P.m	unstable	1	5	11	1	6	6
**OsCNGC3**	217	25402.3	5.81	Cyt/-	unstable	-	4	3	1	3	1
**OsCNGC4**	711	80879.4	9.57	P.m	unstable	2	5	14	-	11	5
**OsCNGC5**	740	84495.7	9.50	P.m	unstable	3	5	8	1	11	2
**OsCNGC6**	724	83367.0	9.14	P.m	unstable	1	4	11	1	11	4
**OsCNGC7**	686	79170.6	9.49	C.t.m	unstable	2	6	12	1	10	5
**OsCNGC8**	629	71828.4	8.15	P.m	stable	2	12	10	3	9	7
**OsCNGC9**	713	82784.1	9.13	P.m	unstable	2	7	10	1	5	5
**OsCNGC10**	692	79762.1	9.14	P.m	unstable	-	6	13	1	6	4
**OsCNGC11**	685	79014.4	9.29	M.i.m	unstable	1	10	14	1	7	2
**OsCNGC12**	772	88353.9	9.08	P.m	unstable	-	9	2	2	7	1
**OsCNGC13**	735	84594.4	9.42	P.m	unstable	-	5	3	1	6	5
**OsCNGC14**	726	81547.0	9.24	P.m	unstable	1	6	7	1	6	2
**OsCNGC15**	666	74381.6	9.71	P.m	unstable	-	5	7	-	16	1
**OsCNGC16**	691	77220.4	9.63	P.m	unstable	-	5	11	1	16	4

MW (Molecular weight), PI (Isoelectric point), cAMP- cGMP (cAMP- and cGMP-dependent protein kinase), Casein (Casein kinase II), PKC (Protein kinase C), Tyr (Tyrosine kinase), N-Myr (N-Myristoylation), N-Glyc (N-glycosylation), P.m (plasma membrane), Cyt (cytoplasm), C.t.m (chloroplast thylakoid membrane), M.i.m (mitochondrial inner membrane).

The subcellular localization of each OsCNGC was predicted by PSORT analysis. Our results evidenced that thirteen OsCNGCs (OsCNGC1-2, OsCNGC4-6, OsCNGC9-10, and OsCNGC12-16) are predicted to be localized in plasma membrane, while the others i.e., OsCNGC3, OsCNGC7 and OsCNGC11 to be in cytoplasm, chloroplast thylakoid membrane and mitochondrial inner membrane, respectively (Table 2). All relevant experiments in the past have indicated that plant CNGC proteins are specifically localized to the plasma membrane [11]. The presence of OsCNGC3 in cytoplasm is not clear, which needs further experimental elucidation. *Arabidopsis* CNGC proteins, when analyzed revealed that 11 out of 20 CNGC proteins were localized in plasma membrane, while the other 9 in chloroplast thylakoid membrane (data not shown).

Translated proteins are often subjected to post–translational modifications (PTMs) to become functionally active. PTMs are the chemical modification of a protein after its translation, and have wide effects on broadening its range of functionality [43]. CNGC channels can be controlled by variations in the phosphorylation state catalyzed by Ser/Thr kinases [44] and phosphatases [45]. When analyzed in ScanProsite, multiple putative phosphorylation sites were revealed in OsCNGC protein sequences, which may act as substrates for several kinases in the form of casein kinase II, protein kinase C, tyrosine kinase and cAMP/cGMP kinases [46]. Francis and Corbin [47] demonstrated that cAMP/cGMP-dependent protein kinases are major intracellular receptors for these nucleotides (cAMP/cGMP), and the actions of these enzymes account for much of the cellular responses to increased levels of cAMP or cGMP.

As a special class of PTMs, several proteins including CNGCs could be covalently altered by a range of lipids, such as myristate and palmitate, a phenomenon known as lipidation [47]. Protein N-myristoylation can alter the lipophilicity of the target protein and facilitate its interaction with membranes thereby affecting its subcellular localization [48–50]. Our bioinformatics analysis revealed that all OsCNGC proteins bear potential N-myristoylation/ N-glycosylation motif sites. The maximum number of N-myristoylation sites is present in *OsCNGC15* and *OsCNGC16* (16), while OsCNGC8 contain highest number (7) of N-glycosylation sites. In mammals, N-myristoylated proteins include protein kinases, phosphatases, guanine nucleotide-binding proteins (such as CNGCs), and Ca^2+^-binding proteins, many of which participate in signal transduction pathways [48, 51, 52]. Similarly, *N*-glycosylation, post-translational modification that occur in many eukaryotic proteins exposed to the extracellular medium (including trans-membrane proteins), is reported to play various roles, including facilitating the folding, trafficking and function of the protein as well as protecting it from an extracellular medium, which, in plants, is acidic and rich in proteases [53]. In a recent study, Meighan et al. [54] demonstrated that extracellular Ca^2+^ and Zn^2+^-dependent proteases (known as matrix metalloproteinase, MMP) increase the ligand sensitivity of rod and cone CNG channels, and prolonged exposure of CNG channels to MMP even caused the channels to become nonconductive due to MMP-dependent proteolysis. They have further indicated that presence of glycosylation sites may defend CNG channels from MMP-dependent proteolysis, and prevent the CNGC channels to become nonconductive [54]. Therefore the PTMs and other sequence-specific features such as N-myristoylation and N-glycosylation in rice CNGCs are likely to be necessary for their proper functioning, localization and regulation (Table 2).

### Motif composition analysis

**Figure 2:**
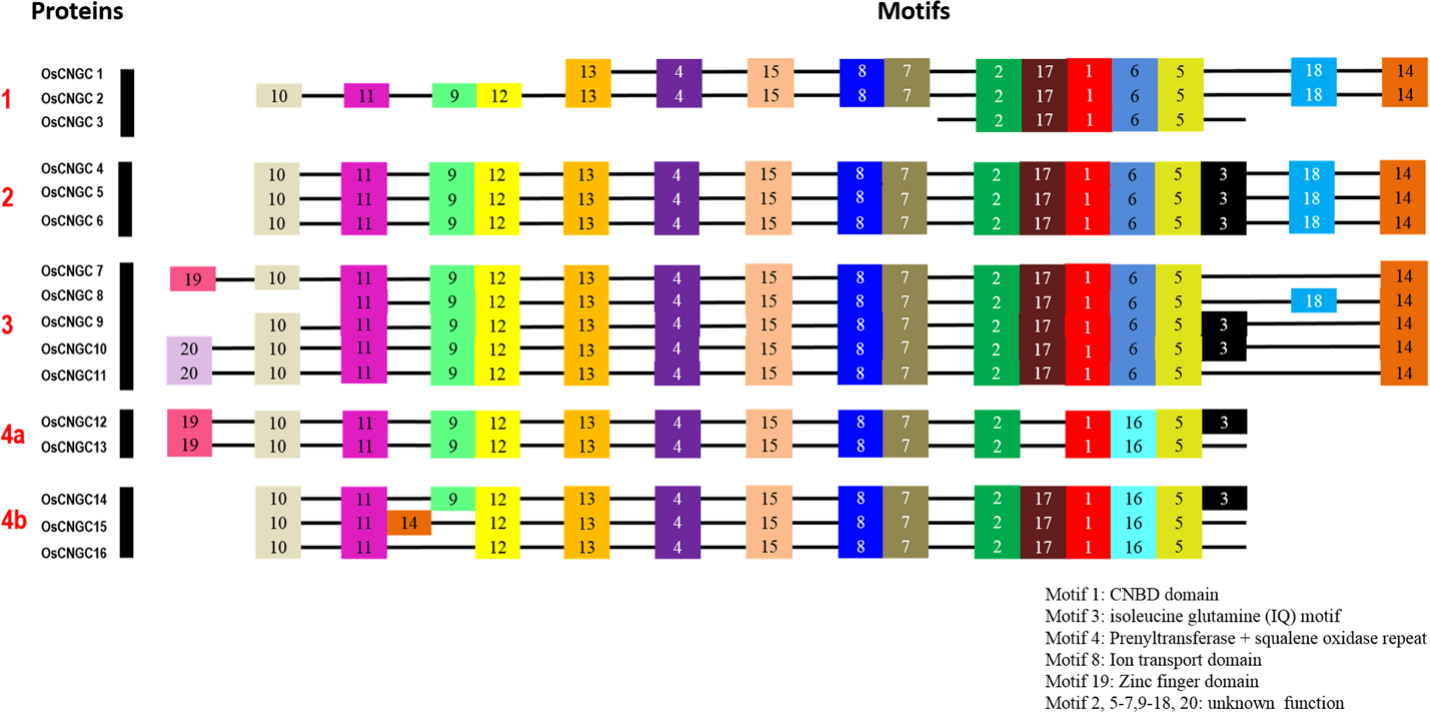
**Distribution of Conserved motifs in rice CNGC proteins identified using MEME search tool.** Schematic representation of motifs composition in OsCNGC protein sequences using MEME motif search tool for each groups given separately. Each motif is represented by a number in colored box. Length of box does not correspond to length of motif. Order of the motifs corresponds to position of motifs in individual protein sequence.

A functional motif-based recognition of CNGC proteins can contribute to understanding the evolutionary history of this gene family including possible role of genome and gene duplication events [55]. Motif search by the MEME/MAST identified 19 conserved motifs in rice CNGC proteins (Figure 2). Among these, five motifs (1, 3, 4, 8 and 19) were identified in rice, and found to be associated with functionally defined domains (Figure 2; Additional files 3 and 4). All the OsCNGC proteins share a conserved binding domain for cyclic nucleotides (CNBD) in the central region, referred to as motif 1. Plant CNGCs are believed to be activated by direct and reversible binding of nucleotides cGMP and cAMP to the CNBD, which allosterically causes the channel to open [19]. In the presence of calcium, CaM binds to the CaMBD and prevents the binding of cyclic nucleotides to the CNBD, and thereby stops the activation of CNGCs by cyclic nucleotides [19, 56]. This competitive regulation by cyclic nucleotides and CaMs was predicted [21] and experimentally verified for *AtCNGC*2 [19]. The CaM-binding site was recently mapped to an isoleucine glutamine (IQ) motif by Fischer et al. [57]. Our results also identified IQ as a functional motif (motif 3) within CaMBD, downstream of the CNBD (Figure 2; Additional file 3). IQ motifs have been investigated in detail in animal proteins, which often bind CaM in a Ca^2+^-dependent manner, while Ca^2+^ induces the displacement of the CaM ligand [58]. Fischer et al. [57] showed that IQ motif is conserved among plant CNGCs, this motif enhances the variability of Ca^2+^-dependent channel control mechanisms featuring the functional diversity within this multigene family. However, in rice, IQ motifs were present in less than 50% of the members of OsCNGC family, mainly in the members of group 2, 3 and 4 (OsCNGC4-6, OsCNGC9-10, and OsCNGC12-14, respectively) (Figure 2), suggesting that other regulators may alter the channel opening and for gating control of plant CNGCs which lack these motifs [57]. Other functional motifs denoted as motif 4, and 8, associated with prenyltransferase and squalene oxidase repeat, and ion transport, respectively, was found to be conserved in all OsCNGC proteins except OsCNGC3. The functions of other motifs identified in OsCNGC proteins 2, 5–7, 9–18, and 20 have not been reported in any plant or animal so far. Moreover, the sequence logo of three potential motifs, CNBD, IQ and ion transport one can determine not only the consensus sequence but also the relative frequency of bases and the information content (measured in bits) at every position in a site or sequence. The logo displays both significant residues and subtle sequence patterns (Additional file 3).

We also performed MEME analysis on 20 *Arabidopsis* CNGC proteins for comparison. The diagram of AtCNGC motifs, their details and the sequence logo of functional motifs is given in Additional files 4, 5, and 6. The numbers of motifs are designated similarly in both rice and *Arabidopsis* CNGC proteins, each corresponding to the position/location of the respective motif. In *Arabidopsis*, only three motifs (motif 1.1, 1.2 and 3) were identified representing the CNBD domain, and IQ motif, where CNBD domain was represented by two motifs (motif 1.1 and 1.2) interrupted by 4 non-functional motifs. Interestingly, motifs associated with ion transport, zinc finger, prenyltransferase and squalene oxidase repeat were absent in *Arabidopsis* (Additional files 5 and 6). Though, individual scan of AtCNGC proteins revealed the presence of ion transport domain in some members (data not shown).

**Figure 3:**
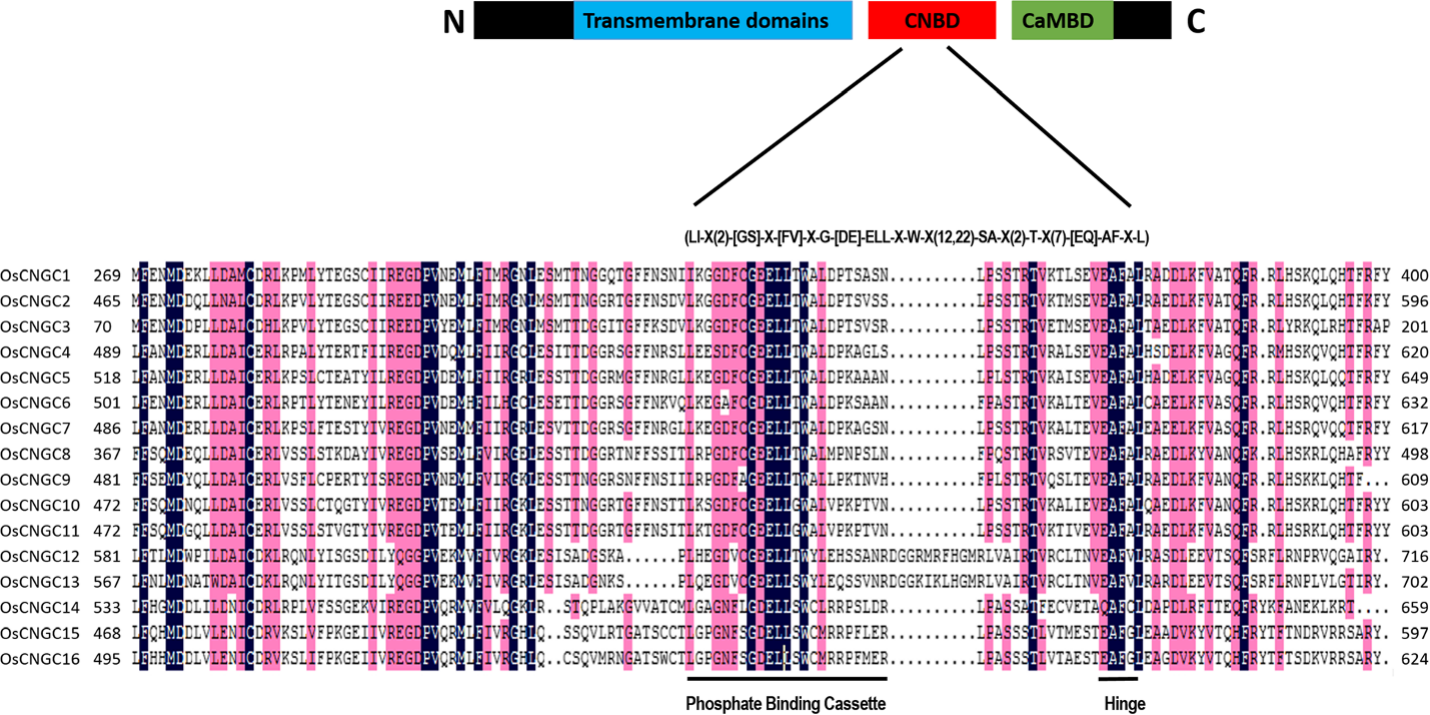
**The rice CNGC-specific motif spans the putative PBC and the hinge within the CNBD of the 16 OsCNGCs.** The diagram at the top represents three regions of plant CNGCs: the six transmembrane domains (TM), a CNBD containing a PBC and the hinge, and CaMBD. The rice CNGC-specific amino acid motif is shown below the cartoon. In the square brackets “[]” are the amino acids allowed in this position of the motif, “X” represents any amino acid and the round brackets “()” indicate the number of amino acids. Below is the alignment of CNBD domains of 16 rice CNGCs. Residues in white highlighted in blue indicate 100% identity among the 16 rice CNGCs.

As mentioned earlier, an essential structural feature of plant CNGCs is their CNBD, a direct binding site for cAMP/cGMP to modify the channel opening [59]. The most conserved feature of CNBD domain is the PBC that makes direct contact with cAMP [60], and the hinge region, essential for the capping of cAMP by the C-helix of the CNBD [61]. No plant CNGC-specific motifs had been reported until Zelman et al. [21] aligned the PBC and hinge regions for 20 AtCNGC proteins, from which the following consensus motif was derived: ([21]-X(2)-[GS]-X-[FYIVS]-X-G-X(0,1)-[DE]-LL-X(8,25)-[1]-X(9)-[VLIT]-E-X-F-[62]). Similarly we aligned the CNBD region of rice CNGCs (Individually and with *Arabidopsis* CNGCs) and identified a putative PBC and a hinge (Figure 3; Additional file 7). We identified a conserved (100%) glycine (G), acidic residue glutamate (E) followed by two aliphatic leucines (L) and aromatic tryptophan (W) inside the PBCs (Figure 3). We also detected that the putative hinge also comprises a conserved (100%) aromatic phenylalanine (F), aliphatic alanine (A) and leucines (L) (Figure 3). A neutral residue, threonine (T) was also found to be conserved (100%) near the hinge region. We successively built a stringent motif: (LI-X(2)-[GS]-X-[FV]-X-G-[DE]-ELL-X-W-X(12,22)-SA-X(2)-T-X(7)-[EQ]-AF-X-L) that recognizes the 16 rice CNGCs specifically. The rice PBC and hinge regions differed from the conserved residues of *Arabidopsis*. For example, residues E, W and T within PBCs, and residues A and L in hinges were found be conserved at 100% in rice CNGCs only. The diversity of motif patterns between the rice and *Arabidopsis* indicate the functional divergence across the species and individual groups.

### Phylogenetic analysis

#### Phylogenetic relationship between rice and *Arabidopsis*CNGC family genes

**Figure 4:**
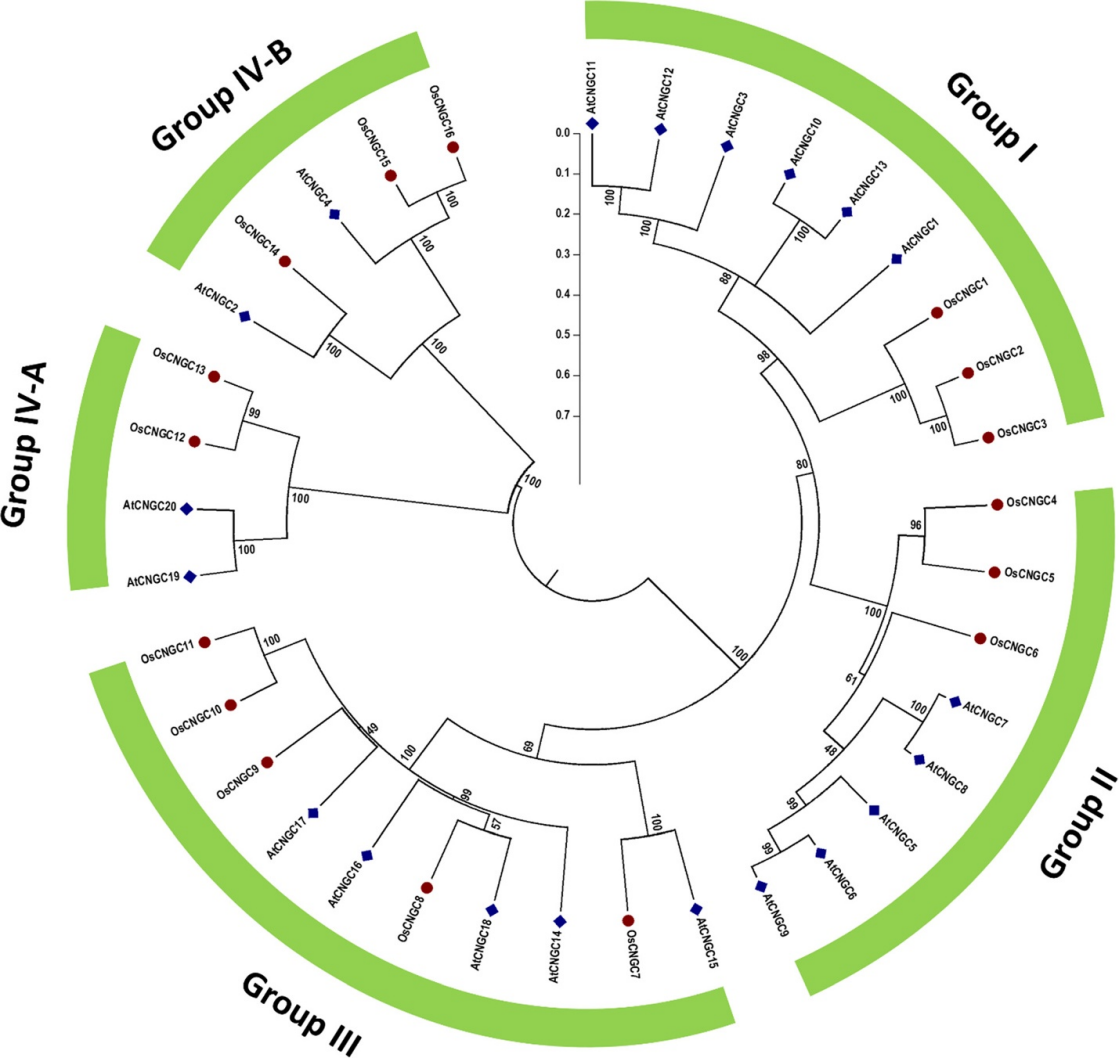
**Phylogenetic tree of OsCNGC and AtCNGC proteins.** The multiple alignment was performed by ClustalX program. MEGA 6.0 was used to create the maximum likelihood (ML) under the Jones-Taylor-Thornton (JTT) model. The bootstrap values from 1000 resampling are given at each node. The rice CNGC genes identified in this study are shown in red circles and *Arabidopsis* CNGCs are shown in blue squares. Rice CNGC genes were designated according to their order in phylogeny.

To determine the phylogenic relationship of CNGC family between rice and *Arabidopsis*, a maximum likelihood (ML) phylogenetic tree was constructed using full-length amino acid sequences. Four groups, as described by Mäser et al. [18] were identified containing representative gene of both rice and *Arabidopsis.* All the *Arabidopsis* CNGC proteins were found to lie in groups similar to those identified previously [18]. Of the four groups, three groups (Group I, II and III) are monophyletic, while one group (IV) is sub-divided into two distinct clades, named group IV-A and IV-B (Figure 4). Group I comprises three members from rice CNGCs (OsCNGC1 to OsCNGC3) and six from *Arabidopsis* (AtCNGC1, 3, 10, 11, 12 and 13)*.* Similarly, Group II contains three rice CNGCs (OsCNGC4 to OsCNGC6) and five AtCNGCs (AtCNGC5 to AtCNGC9). However, Group III embraces five in rice (OsCNGC7 to OsCNGC11) and five in *Arabidopsis* (AtCNGC14 to AtCNGC18), thus form the largest group, with 10 members. Two CNGCs from each rice (OsCNGC12 and OsCNGC13) and *Arabidopsis* (AtCNGC19 and AtCNGC20) were assigned to group IV-A, while, three rice CNGCs (OsCNGC14 to OsCNGC16) and two *Arabidopsis* CNGCs (AtCNGC2 and AtCNGC4) segregated into group IV-B (Figure 4). A separate phylogenetic tree was also generated from conserved CNBD domains sequences of all the CNGC proteins in rice and *Arabidopsis*, which resulted into similar clustering pattern.

During the evolution of a gene family, gene duplication plays important roles in generating new members and creating novel gene functions, hence increasing numbers and diversifying functions of genes within a gene family [63, 64]. The phylogenetic analysis between rice and *Arabidopsis* following previously described model [64, 65] demonstrates the evolutionary trend of gene duplication in both taxa.

Phylogenetic data provide a framework for making more appropriate intergenomic comparisons, by determining whether chromosomal duplications within taxa pre-date or post-date divergence among taxa [65]. The current analysis shows that divergence of two taxa may have pre-dated the gene duplication events among them resulting in gene expansion within individual group [63] (Additional file 8). This is further strengthened by our detailed phylogenetic analysis among all plant lineages given ahead. The detailed phylogeny also points towards the duplication of CNGC genes occurring after speciation and after divergence of angiosperms into mono and dicots. Interestingly AtCNGC2 and OsCNGC14; AtCNGC15 and OsCNGC7 are not in congruence with the pattern of other sister groups. The existing anomaly between these taxa could not be reasoned out.

### Phylogenetic analysis of the *CNGC*genes in all plant lineages

For a better understanding of the evolutionary history of the *CNGC* gene family, we comprehensively analyzed the phylogeny and evolution of the *CNGC* gene family in all plant lineages. We used the amino acid sequences of rice and *Arabidopsis* CNGCs to query the OrthologDB, Gramene, TAIR, ConGenIE, NCBI, Phytozome and RGAP databases of plant species (see Materials and Methods). In order to obtain only specific *CNGC* sequences, we removed the hits with exact duplicates (same identifiers, same sequence and different identifiers, same sequence), annotated fragments, as well as any unrelated gene like AKT or KAT family channels from subsequent analyses (Additional file 9). We identified 235 CNGC-like sequences from 32 different species including green algae (8), bryophytes (4), lycophytes (4), gymnosperms (3), monocots (58) and dicots (158) [66, 67], whose protein sequences harbored typical domains and motifs of CNGC proteins, i.e. CNBD (essential), ion transport (optional) and IQ (optional). The copy number of *CNGC* gene family ranged from 1 to 28 amongst the species, where, *Glycine max* had the largest *CNGC* gene family containing 28 genes (Additional file 9). However, it should be indicated that the single *CNGC* in wild banana (*Musa acuminata*) may not reflect the true number of *CNGC* genes in this species; rather it is due to the limited availability of its genomic information.

**Figure 5:**
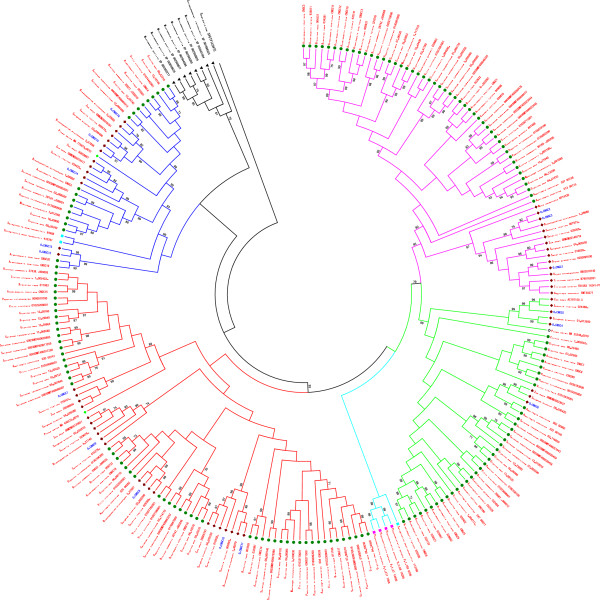
**The maximum-likelihood (ML) phylogenetic tree of** ***CNGC*** **family genes in 235 representative plant species.** The rooted maximum-likelihood (ML) phylogenetic tree was inferred from the amino acid sequences alignment of full length proteins using MEGA 6.0, under the Jones-Taylor-Thornton (JTT) model. The bootstrap values from 1000 resampling, and cut-off values >70% are given at each node. The node color specifies group i.e. pink = I, light green = II-A, aqua = II-B, red = III, blue = IV-A, and black = IV-B. Rice CNGC genes are shown in blue color. Plant lineages are shown by shapes with different colors, i.e. green circle = dicots, maroon diamond = monocots, green diamond = gymnosperms, Aqua blue square = lycophytes, pink square = moss, and black triangles = green algae.

A maximum likelihood tree was generated using amino acid sequences of the deduced full-length peptides with JTT (Jones, Taylor and Thornton) model. We used SYG1 from *Saccharomyces cerevisiae* as out-group [68]. According to the tree’s topology, *CNGC* gene family of all plant lineages clustered into four distinct groups (I-IV) with significant bootstrap values (Figure 5)*.* Group II and group IV are subdivided into II-A/II-B and IV-A/IV-B. All the *CNGCs* from algae formed the basal lineage and clustered into same group (group VI-B), whereas those from the land plants grouped into several other groups (I–IV), showing that the *CNGC* family originated earlier than the separation of green algae and the ancestor of land plants. Among these four groups, only Group II was conserved in all land plants, showing that all *CNGC* genes from land plants shared a mutual ancestor after the divergence from aquatic plants. Group II-B was solely present in lower land plants including mosses (bryophytes) and lycophytes. Similarly, *CNGCs* from group I expanded in both mono- and dicotyledonous angiosperms, while group II-A and III was prevalent in all embryophytes (angiosperms and gymnosperms) (Figure 5). Group IV-A contain *CNGCs* from vascular plants i.e., embryophytes and lyophytes. These analyses revealed that all *CNGC* genes from land plants originated from a common ancestor, earlier than the split between lower and higher land plants [69], while the lineage specific expansion and divergence happened in higher land plants, particularly in dicots, after diversification from lower land plants, which lead to generation of group II-B in basal land plant and group I-IV in higher land plants. Moreover, *CNGC* genes from similar lineage, such as mosses, lycophytes, gymnosperms and angiosperms, inclined to be clustered together. Our interpretations are corroborated by the findings of previous studies [66, 68, 70, 71].

### *In silico*prediction of potential cis-acting regulatory elements

The cis-acting regulatory elements are important molecular switches involved in the transcriptional regulation of genes during biotic and abiotic stress responses, and may be induced through independent signal transduction pathways [72]. The 5′ upstream noncoding sequences are not well conserved among the *OsCNGC* genes (data not shown), signifying that these genes may be differentially expressed in response to various stimuli, even if they encode identical proteins [34]. To obtain a preliminary clue about the regulatory function and the molecular mechanism of *OsCNGC* genes in transcriptional regulation, potential cis-acting regulatory elements in upstream (1000 bp) sequences of the *CNGC* genes were analyzed. Results exhibited that *OsCNGC* gene promoter sequences contain numerous cis-acting regulatory elements regulatory sites for hormones, different biotic and abiotic factors (Table 3; Additional file 10). Twenty different cis-acting regulatory elements are regulated by ABA; among them, MYB recognition site (MYB1AT) related to the drought- and ABA-induced gene expression [73], is conserved among 13 *OsCNGC* genes. Moreover, only 4 elements (ERELEE4, LECPLEACS2, GCCCORE and AGCBOXNPGLB) are regulated by ethylene, two elements (ARR1AT, CPBCSPOR) are regulated by cytokinin (Additional file 10). The patterns of cis-acting regulatory elements differed significantly among the *OsCNGC* genes. For example, the promoter of nine *OsCNGCs* (2, 6, 8, 9 and 11–15) contain elements that may respond to all these hormones, biotic and abiotic stress, while others lack the elements responsive to ethylene and cytokinin (Table 3). Comparatively, only three elements were found to be conserved and consistently present in the upstream region of all 16 *OsCNGCs*, i.e. two abiotic stress signaling related elements, consensus GT-1 binding site (GT1CONSENSUS) and MYC recognition site (MYCCONSENSUSAT), and WRKY transcription factor (WRKY71OS). The WRKY transcription factor (WRKY71OS) is reported as binding site of rice WRKY71, a transcriptional repressor of the gibberellin signaling pathway [74], and a core of TGAC-containing W-box in WRKY1, which plays an important role in the regulation of early defense-response genes in rice. The presence of most these cis-acting regulatory elements in *OsCNGC* gene promoters depicts their importance in regulation of this gene family (Table 3).

**Table 3:** **Total number of cis-acting regulatory elements in** ***OsCNGC*** **gene family**

Regulators	***OsCNGCs***															
	***1***	***2***	***3***	***4***	***5***	***6***	***7***	***8***	***9***	***10***	***11***	***12***	***13***	***14***	***15***	***16***
**ABA**	6	6	8	3	10	3	5	14	7	9	4	9	7	14	6	10
**Auxin**	5	6	3	3	9	4	1	1	7	7	6	1	3	6	3	10
**Ethylene**	-	2	-	11	-	2	-	9	1	-	4	9	1	2	1	-
**Cytokinin**	9	7	11	-	5	8	8	6	8	7	13	11	9	7	6	9
**Biotic**	19	14	21	4	14	19	4	11	17	25	14	12	14	14	10	20
**Abiotic**	50	42	49	35	28	34	35	50	53	34	26	48	45	63	36	45

### Expression analysis of *OsCNGCs*

Plant *CNGC* genes have been implicated in diverse aspects of plant growth and development, including responses to hormonal, abiotic and biotic stress [75]. In past, many plant mutants of *CNGCs*, and transgenic plants expressing full-length or mutant *CNGCs*, were characterized to explore their role in plants [16]. These researches mainly focused on *Arabidopsis CNGCs* and found that *CNGC* mutants exhibit various phenotypes and multi-functional behaviors. Briefly, *AtCNGC1* are probably involved in Ca^2+^ uptake into plants [76], *AtCNGC*2, 4, 11 and 12 in plant defense and disease resistance pathway [77], *AtCNGC*3 in homeostasis [24, 78], and *AtCNGC*2, 4, 7, 8, 10, 16, and 18 in plant development [79–81]. To obtain information about potential differential gene functions in rice, we employed bioinformatics and experimental approaches to assess the role of *OsCNGCs* in plant development, and expression patterns in response to various stress.

### Expression of *OsCNGCs*in development

The expression profile of rice leaves obtained from public database RiceXPro exhibited that most of *OsCNGC* genes showed their maximum expression in late vegetative and early reproductive stage (from day 41 to day 62) during life cycle of rice in field conditions, while, *OsCNGC5* had its highest expression level at early vegetative stage. Overall, highest expression was observed for *OsCNGC14* at 118th day during late reproductive stage, while lowest expression was noted for *OsCNGC8* at 20th day of rice growth in field conditions (Additional file 11).

### Expression of *OsCNGC*s in rice seedlings

**Figure 6:**
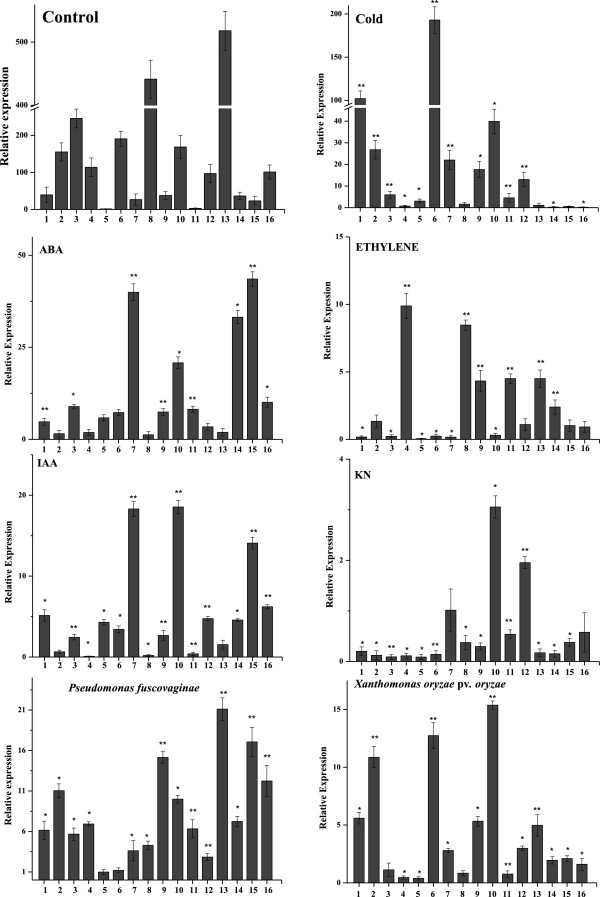
**Expression profiles of OsCNGC genes in 4 weeks old rice leaves.** The expression patterns of OsCNGC genes under hormonal (ABA, ETH, IAA, and KN), biotic (*Xoo* and *P. fuscovaginae*) and abiotic (cold) stresses are depicted in figure. Gene expression was perforemd by qPCR, 4 h after each treatment and non-treated CK. The Y-axis indicates the relative experssion levels (in folds) of treated *versus* untreated (control) for each gene and X axis shows OsCNGC genes. The error bars were calculated based on three biological repliactes using standard deviation. *significant difference at p < 0.05, **means very significant difference p < 0.01.

By using quantitative real-time PCR (qPCR) analysis, the expression level of *OsCNGC* genes was investigated in leaves of the rice cultivar Nipponbare (*O. sativa* L. ssp. *japonica*). The results indicated there were tremendous variations among the *OsCNGC* genes (Figure 6). *OsCNGC8* and *13* were highly expressed and had transcripts abundances of 441 and 518 times that of *OsCNGC5*, while *OsCNGC11* had the lowest transcripts abundances (Figure 6).

### Response to hormonal stresses

To examine the expression pattern of *OsCNGCs* in response to different plant hormones, qPCR analysis were performed using the total RNA extracted from leaves of the rice cultivar Nipponbare (*O. sativa* L. ssp. *japonica*), subjected to ABA (abscisic acid), ETH (ethylene), IAA (indole acetic acid) and KN (kinetin). Primers used for RT-PCR analysis are listed in Table 4.

**Table 4:** **List of** ***OsCNGC*** **and internal control genes primers used for qPCR gene expression**

Names of primers	Sequence(5′to3′)	Product Length
***OsCNGC*** **1-F**	AGTAATGCAGCTAGAAATAAC	207
***OsCNGC*** **1-R**	CAAAACTAGACCTGATGTCGA	
***OsCNGC*** **2-F**	GTATGCAAATGTCGCCACCC	158
***OsCNGC*** **2-R**	GCCTCCGTGGTGACATAACT	
***OsCNGC*** **3-F**	GCTCAACCAGGACAGTGGAG	134
***OsCNGC*** **3-R**	CAAAGGGGCCCTAAACGTG	
***OsCNGC*** **4-F**	GCGGCAGTACATCAACGAGAC	228
***OsCNGC*** **4-R**	AACGTCGCCAGGAATATCGA	
***OsCNGC*** **5-F**	CAGTACCGGTGGCTGGAGAC	191
***OsCNGC*** **5-R**	TCCGTGCACAGGCTGGGCTT	
***OsCNGC*** **6-F**	TTCTGCGCACAAAGCTCAAT	121
***OsCNGC*** **6-R**	GCTAAACTTCAGGGTGCTCCT	
***OsCNGC*** **7-F**	TCTTCCTCTACCTCACGGGG	213
***OsCNGC*** **7-R**	GGTACCTCCTGGCGATCTTG	
***OsCNGC*** **8-F**	ATCCGCTACCTCAAGAACGA	118
***OsCNGC*** **8-R**	TCGAGAGCGTGTTGTTGGTG	
***OsCNGC*** **9-F**	TCCGGTTCTACTCCCACCAT	137
***OsCNGC*** **9-R**	TGGATGTTCTTCTGGCCACC	
***OsCNGC*** **10-F**	GTTTCTGCTTCCTCCAGGCT	162
***OsCNGC*** **10-R**	AGCGAGCAAGAACTCGTCAA	
***OsCNGC*** **11-F**	CATGGGCCGCTTGTTTCATC	161
***OsCNGC*** **11-R**	GGCTCCTTTCCTGACTGCAT	
***OsCNGC*** **12-F**	CACCAGATATGCCTCCTCGA	233
***OsCNGC*** **12-R**	ACTGTCCAATGTACTTGATC	
***OsCNGC*** **13-F**	CTCGATAAGCTGCCTCGTCCA	217
***OsCNGC*** **13-R**	ACGGAGCTAAACTTCTCTGG	
***OsCNGC*** **14-F**	TACAAGGCAAGGACGACGAC	166
***OsCNGC*** **14-R**	CACTCTCGGTCCCAATCCAC	
***OsCNGC*** **15-F**	TGTGCAGAGGTTATGGCTGG	192
***OsCNGC*** **15-R**	GACACGGCGTACAGGAAGAG	
***OsCNGC*** **16-F**	GCACTTCCGGTACACCTTCA	254
***OsCNGC*** **16-R**	AGAAGTCGTCGTCCTGGTTG	
**18S rRNA-F**	TGTGCCGCTAGAGGTGAAATT	65
**18S rRNA-R**	TGGCAAATGCTTTCGCTTT	
**UBQ5-F**	ACCACTTCGACCGCCACTACT	69
**UBQ5-R**	ACGCCTAAGCCTGCTGGTT	

The expression of several CNGC genes in rice is regulated by plant hormones. In general, more genes were up-regulated by ABA (12) and IAA (11) as compared to KN (2) and ETH (6). In sharp contrast, twelve and 6 genes were significantly down-regulated by KN and ETH, respectively, while none and 3 genes were down-regulated by ABA and IAA, respectively (Figure 6). The CNGC genes belonging to different phylogenetic groups showed different expression levels from each other, depending on the treatment type, while the genes in the same group showed a similar expression pattern. For example, the expression of *OsCNGC7* and *OsCNGC10* (belonging to same phylogenetic group III) was simultaneously up-regulated by all but ETH treatments (Figure 6). It has been proposed that the proteins classified in the same classes may have similar functions [82, 83]. Similar conclusion has been drawn by Ma et al. [84], while studying the expression of the C3HC4-type RING finger gene family in rice.

### Response to biotic and abiotic stress

To determine *OsCNGCs* role in disease resistance, we quantified gene expression levels in 4 weeks old rice seedlings, inoculated with two phytopathogens, *Xanthomonas oryzae* pv. *oryzae* (*Xoo*) and *Pseudomonas fuscovaginae*, that cause a serious blight and bacterial sheath brown rot of rice, respectively. The expression of the *OsCNGC* family genes at 4 hpi (hour post-inoculation) are shown in Figure 6. The data depicted that large number of *OsCNGC* genes were significantly up-regulated after infection with each pathogen within 4 h. Fourteen *OsCNGCs* were significantly up-regulated by *P. fuscovaginae* inoculation with *OsCNGC13* being at the highest relative level of expression. The expression of *OsCNGC5 and 6* were not affected (Figure 6). Similarly 11 *OsCNGCs* were up-regulated by *Xoo*, where the expression of *OsCNGC6* and *10* increased by 12.5 and 15.3-folds respectively. Previous genetic approaches have identified three *CNGC*s in *Arabidopsis* (*AtCNGC2* and *4*) exhibited alterations in defense responses and reported to be involved in plant immunity [14]. These two mutants are reported to have successfully managed the lower growth rates of *P. syringae,* accomplished by sustaining high levels of salicylic acid, leading to constitutive expression of pathogenesis-related (PR) genes, and other defense responses [85]. It is noteworthy to mention that AtCNGC*2 & 4* are the closest homologue of the members of rice *CNGCs* from group IV, which showed highest expression levels in response to *P. fuscovaginae,* suggesting that these *OsCNGC* genes are early responsive and sensitive to biotic stress, and their regulation plays an important role in plant defense.

Similarly, the expression levels of the *OsCNGC* family genes under cold stress condition were also investigated. The results indicated that 10 *OsCNGCs* were up-regulated under cold stress. It was noted that the expression of *OsCNGCs* belonging to phylogenetic group I, II and III were significantly induced by cold stress, with fold range of 5–102 for group I (*OsCNGC1-3*), 3–192 for group II (*OsCNGC5* and *6*), and 4–39 folds for group III (*OsCNGC7-11*), respectively (Figure 6). On other hand, *OsCNGCs* from group IV (*OsCNGC14-16*) showed relatively lower expression under cold stress. Overall, *OsCNGC*6 gene showed the highest expression level (192-folds increase) in response to cold stress, while *OsCNGC16* showed the lowest level expression (−2-fold decrease) (Figure 6).

Interestingly, six of 16 gene pairs located on duplicated chromosomal segments showed differential expression patterns, suggesting that most duplicated gene pairs were under the diverse transcriptional controls. For example, *OsCNGC1* and *OsCNGC2* localized on duplicated segments exhibit similar expression patterns in response to both pathogenic and abiotic stresses (ABA & IAA), indicating their overlapping functions. This finding was similar to previous results showing that the expression of duplicated genes frequently diverges compared to that of their ancestors, suggesting that duplication was a major reason for the enrichment of the functions of this family during the long course of evolution [63, 86, 87]. It is proposed that a change in a duplicated locus of two genes might not exhibit the same morphological and/or physiological phenotypes. However, when gene duplication occurs, some genes may retain their original functions and expression patterns [22]. No close correlation was observed between cis-acting regulatory elements in upstream sequences of rice CNGC genes and their qPCR expression levels. It is probable that the differential response of genes to different stimuli is due to different upstream sequences and introns. These results suggest that many of the *OsCNGC* genes play discrete roles in development, response to stress and plant defense related functions in rice. However, further studies based on gene knockout techniques are required to properly identify and explore their functions.

## Conclusions

There are 16 *CNGC* genes in rice, which are classified into 4 groups (I-IV) and two sub-groups (IV-A and IV- B); this gene family appears to have expanded through both chromosomal segmentation and tandem duplications. The *CNGCs* from all plant lineages are also clustered into four groups, with group II being conserved in all land plants. All the OsCNGC protein sequences contain a CNGC specific domain CNBD that comprises a PBC and a “hinge” region, featured by a stringent motif: (LI-X(2)-[GS]-X-[FV]-X-G-[DE]-ELL-X-W-X(12,22)-SA-X(2)-T-X(7)-[EQ]-AF-X-L). Various cis-acting regulatory elements were identified in the upstream sequences present on both positive and negative strands. In addition, the genes transcripts significantly respond to multiple stimuli, as demonstrated by their expression patterns to exogenous hormonal (abscisic acid, indoleacetic acid, kinetin and ethylene), biotic (*P. fuscovaginae* and *Xoo*) and abiotic (cold) stresses.

## Methods

### Bioinformatics analysis

#### Identification of CNGC genes in rice

To identify members of CNGC gene family, multiple database searches were performed. The *Arabidopsis* CNGC (*AtCNGC*s) gene sequences obtained from the TAIR database [88] were used as queries to perform repetitive blast searches against MSU Rice Genome Annotation Project database (RGAP/ RAP-DB) [89]. Additionally, all protein sequences were then used as queries to perform multiple database searches against proteome and genome files downloaded from these databases. Stand-alone versions of BLASTP and TBLASTN available from Basic Local Alignment Search Tool [90] were used with the e-value cutoff set to 1e-003. All retrieved non-redundant sequences were collected from phytozome database v9.1 [91], and subjected to domain analysis by using six different domain analysis programs: the Pfam 27.0 [92], CDD [93], SMART [94], PROSITE profiles [95], supfam [96] and Gene3D [97], with the default cut off parameters. Genes without CNGC-specific CNBD domains or having a size of below < 200 amino acids domains were rejected.

### Chromosomal localization and gene duplication

Positional information on the rice *CNGC* genes was provided by the rice genome databases, RGAP and GRAMENE [98]. Rice TOGO Browser was used to locate each gene on a chromosome [99]. The Plant Genome Duplication Database (PGDD) was utilized to analyze the duplication of each gene with a maximum distance of 500 kb permitted between collinear genes [100].

### Prediction of gene structure and cis-acting regulatory elements

The gene structure (Exon-intron distribution) analyses of the *CNGC* families of rice and *Arabidopsis* were carried out using the Gene Structure Display Server (GSDS) with default settings [101].

Promoter sequences (−1000 bps) of *OsCNGC* family genes were obtained from the RGAP database. The upstream 1000 bp sequence of *CNGC* genes were searched for a variety of cis-acting regulatory elements by ‘Signal Scan Search’ program in the PLACE database [40].

### Prediction of rice CNGC protein sequence features

Protein sequences of putative OsCNGC members collected from the RGAP database were analyzed using ExPASy proteomics Server [102]. The information in number of amino acids, isoelectric points, molecular weights and instability index were obtained. The proteins having instability index of >40 were considered as unstable. The cellular localization of each CNGC protein was identified using the PSORT online program [103]. The post-translational modifications such as phosphorylation sites casein kinase II, protein kinase C, tyrosine kinase, cAMP- and cGMP-kinase, N-Myristoylation, N-Glycosylation, were predicted at ScanProsite tool [104].

### Phylogenetic analysis of CNGC genes in *Arabidopsis*and rice

To identify the number of groups formed by rice *CNGC* genes in comparison to *Arabidopsis*, the amino acid sequences of full length of 20 CNGCs from *A. thaliana* and 16 CNGCs from *O. sativa* were aligned by using ClustalX 2.01 program [105] with default settings. The MUSCLE (Multiple Sequence Comparison by Log-Expectation) program (version 3.52) was also used to perform multiple sequence alignments to confirm the ClustalX data output [106]. A complete phylogenetic analysis between rice and Arabidopsis was also carried out. Individual protein sets were aligned using MAFFT v7.017 with the L-INS-i model [107]. The protein sequence of outgroups *Amborella trichopoda* and *Selaginella moellendorffii* was downloaded from Amborella Genome Database [108] and Phtyozome [91] respectively. The alignment was further used to construct a maximum likelihood Newick-formatted phylogenetic tree by FastTree [109] keeping the default settings.

Furthermore, we carried out phylogenetic investigation of CNGCs in all plant lineages to comprehend the evolutionary patterns followed by these genes. For this purpose, the amino acid sequences from Arabidopsis CNGCs were used as queries against GRAMENE [98], OrthologDB [110], Phytozome [92], UniProt [111], MSU-RGAP [87], TAIR [88], ConGenIE [112] and GeneBank [90] of plant. These sequences were subsequently submitted to Pfam for analysis and confirmation of CNGC-specific functional domain [92]. Afterwards, 235 typical identified CNGC proteins were subjected to multiple alignments using MUSCLE (version 3.52) as mentioned above.

Finally, phylogenetic trees were constructed based on alignments using maximum likelihood (ML) method of MEGA 6.0 (Molecular Evolutionary Genetics Analysis) with complete deletion option parameters engaged [113]. The reliability of the trees was tested using bootstrapping with 1000 replicates. Then, to build the condensed tree it was selected a cut-off value equal to 50%, where the values >70% are displayed on each clade. Images of the phylogenetic trees were also drawn using MEGA 6.0.

### Motif composition analysis of OsCNGC proteins

The conserved protein motifs of all CNGC protein sequences from the rice and *Arabidopsis* were analyzed using the MEME 4.6.1 and MAST motif search tool [114] with the following parameters: number of different motifs as 10, minimum motif width as 6 and a maximum motif width set to 50. The functional annotation of these motifs was analyzed by Pfam program.

Moreover, the CNBD domain regions of 20 *Arabidopsis* and 16 rice CNGC proteins were aligned to construct rice CNGC specific motif. First, the amino acid sequences of all 36 CNGCs were subjected to Pfam database, to determine the position of CNBD regions. Alignments were performed using the default parameters of ClustalX (version 1.83) and subsequently examined and edited in GeneDoc [115]. Finally, a detailed diagram was constructed for in depth sequence analysis.

### Experimental verification

#### Plant materials for expression analysis

Rice seeds of Nipponbare (*O. sativa* L. ssp. *japonica*) were surface-sterilized with 3% sodium hypochlorite for 30 min, germinated at 28°C for 3 days, and then grown in nutrient solution under controlled conditions of 14 h light 30°C/10 h dark 22°C and 70% relative humidity. The rice seedlings at the three-leaf stage (approx. 4 weeks old) were subjected to different stresses. For hormonal treatment, rice leaves were sprayed with six different hormones including 100 μM ABA, 10 mM ETH, 100 μM IAA, 100 μM KN and sterilized water as control. For cold stress treatment, seedlings were incubated at 4°C for 4 h.

For pathogen inoculation, the bacterial pathogen *P. fuscovaginae* and *Xoo* were incubated overnight at 28°C on LB medium plates containing rifampicin (50 μg/mL) and kanamycin (50 μg/mL), inoculated on LB broth. The bacterial cells were collected by centrifugation and then diluted into suspensions to a concentration of OD600 = 0.002 and 0.5, using 10 mM of MgCl_2_ buffer or sterilized ddH_2_O, respectively. The prepared bacterial solution was infiltrated into leaves of 4 weeks old rice plants. Sterile MgCl_2_ buffer or sterilized ddH_2_O was served as controls. A pool of leaves from ten rice seedlings were collected as one biological replicate, and each stress treatment was repeated three times. The samples were placed in liquid nitrogen immediately after sampling and stored at −80°C for total RNA isolation.

### Gene expression analysis with quantitative real-time PCR

Samples were ground in liquid nitrogen using a mortar and pestle. Total RNA (4 mg) was isolated using RNAiso (Takara) and treated with RNase-free DNase I (Takara) for 15 min to eliminate possible contaminating DNA. The resulting cDNA was reverse transcribed using the PrimeScript RT regent kit (TAKARA, Japan) and used for gene expression analysis through qPCR. The qPCRs were conducted in Step One Real-Time PCR System (Applied Biosystems, USA) using SYBER Premix Ex Taq reagents (TAKARA, Japan) following the program: 95°C for 30 seconds, 95°C for 5 seconds, and 60°C for 45 seconds for 40 cycles. Relative gene expression values were calculated using the 2-△△Ct method [116], based on the data from three biological replicates for each treatment. To normalize the samples variance, 18S rRNA and Ubiquitin 5 (UBQ5) genes served as internal control, as recommended by many researchers [117–120]. The experiments were conducted three times, each containing three replicates for all genes. For the statistical analysis of the gene expression data, ANOVA (analysis of variance) analysis was performed with SAS software Version 9.2 [121]. The resultant expression profiles are plotted in the form of graphs (Figure 6; Additional file 12).

Furthermore, the CDs of all *CNGC* genes were subjected to Rice XPro microarray expression database [122] to obtain microarray data derived from leaves at various stages of development under natural field conditions.

## Electronic supplementary material

Additional file 1: **Schematic diagram representing structures of OsCNGC genes.** Exons and introns are indicated as green boxes and black lines, respectively. Intron phase numbers 0, 1 and 2 are also shown at the beginning of the introns. The diagram is drawn to scale. The accession numbers for *OsCNGC* genes are listed in Figure 1. (PNG 108 KB)

Additional file 2: **Schematic diagram representing structures of CNGC genes of** ***Arabidopsis.*** Exons and introns are indicated as black boxes and black lines, respectively. Intron phase numbers 0, 1 and 2 are also shown at the beginning of the introns. The diagram is drawn to scale. The accession numbers for *AtCNGC* genes are listed in Additional file 9. (TIFF 742 KB)

Additional file 3: **Sequence LOGOs for each motif of CNGC domains using the MEME algorithm.** Motif 1: CNBD; Motif 3: IQ Motif 8: Ion transport. MEME motifs are displayed by stacks of letters at each position. The total height of the stack is the “information content” of that position in the motif in bits. The height of the individual letters in a stack is the probability of the letter at that position multiplied by the total information content of the stack. X- and Y-axis represents the width of motif and the bits of each letters, respectively. The details of motifs are given in Additional file 4. (PNG 621 KB)

Additional file 4: **Comparison among motifs and domains of OsCNGC and AtCNGC proteins.** OsCNGC protein motifs markedly differ from ATCNGCs. OsCNGC proteins contain a zinc finger domain in addition to conserved CNBD domains present in both taxa. (XLSX 11 KB)

Additional file 5: **Distribution of Conserved motifs in Arabidopsis CNGC proteins identified using MEME search tool.** Schematic representation of motif composition in AtCNGC proteins sequences using MEME motif search tool for each groups given separately. Each motif is represented by a number in colored box. Length of box does not correspond to length of motif. Order of the motifs corresponds to position of motifs in individual protein sequence. (TIFF 649 KB)

Additional file 6: **Sequence LOGOs for each motif of** ***Arabidopsis*** **CNGC domains using the MEME algorithm.** Motif 2: IQ; Motif 3 & 8: CNBD. MEME motifs are displayed by stacks of letters at each position. The total height of the stack is the “information content” of that position in the motif in bits. The height of the individual letters in a stack is the probability of the letter at that position multiplied by the total information content of the stack. X- and Y-axis represents the width of motif and the bits of each letters, respectively. (PNG 597 KB)

Additional file 7: **Multiple alignment profile of the CNBD domains of rice and Arabidopsis CNGCs obtained with ClustalX program.** All the sequences show high level of amino acids conservation. Gaps (dashes) have been introduced to maximize the alignments. The most conserved feature of CNBD domain, the PBC and the hinge region are shown. The numbers at each end of the sequence show the start and stop positions of the region obtained from full length 36 CNGC protein. Followed by the names are the Residues highlighted in black indicate >100% conservation among the 36 CNGCs. Red highlighted residues indicates >70% identity. (PNG 1 MB)

Additional file 8: **Phylogenetic tree of CNGC genes belonging to rice and** ***Arabidopsis.*** A multiple sequence alignment was performed by MAFFT v7.017 with the L-INS-i model. The alignment was further used to construct a maximum likelihood phylogenetic tree by FastTree keeping the default settings. (PDF 11 KB)

Additional file 9: **List of CNGC-like genes identified in all green plant lineages.** (XLSX 16 KB)

Additional file 10: **List of all cis-acting regulatory elements in 16** ***OsCNGCs.*** (XLSX 14 KB)

Additional file 11: **A global expression profiles of rice CNGC genes at various stages of development.** The data is obtained from RiceXPro [119]. The field/development datasets correspond to microarray data derived from leaves at various stages of development under natural field conditions. Each column represents the data of one replicate, as given in the database. (XLSX 565 KB)

Additional file 12: **Expression profiles of OsCNGC genes in 4 weeks old rice leaves with UBQ5 internal control.** Gene expression was perforemd by qPCR, 4 h after each treatment and non-treated CK. (A) Expression in response to different hormonal treatments. (B) Expression in response to pathogens inoculation with *Xoo* and *P. fuscovaginae* (biotic stress). (C) Expression in response to cold (abiotic stress). (DOCX 408 KB)
