# Supplementary figures and images for: Cyclic nucleotide-gated ion channel gene family in rice, identification, characterization and experimental analysis of expression response to plant hormones, biotic and abiotic stresses
Source: BMC Genomics. 2014 Oct 4;15(1):853. doi: 10.1186/1471-2164-15-853 (PMC4197254; doi:10.1186/1471-2164-15-853)

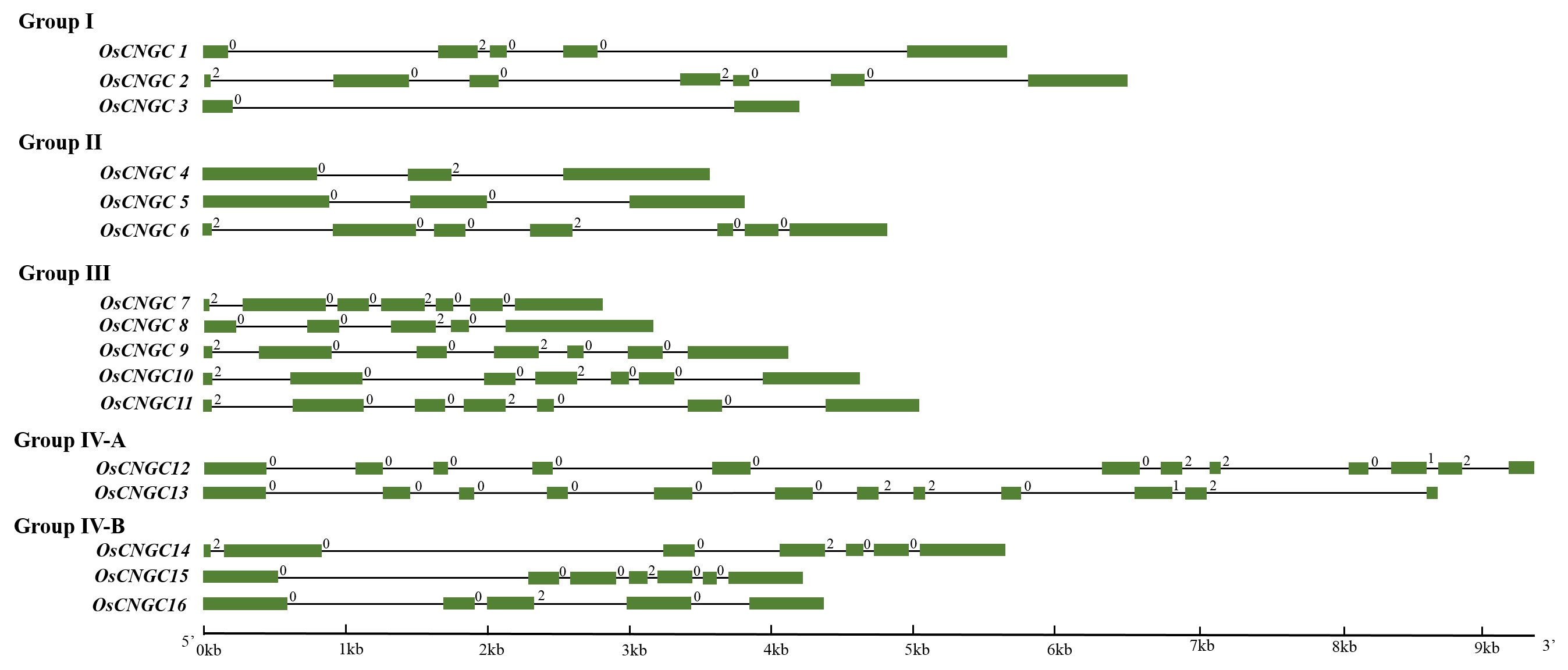

Supplement: Supplementary file 1 — Additional file 1: Schematic diagram representing structures of OsCNGC genes. Exons and introns are indicated as green boxes and black lines, respectively. Intron phase numbers 0, 1 and 2 are also shown at the beginning of the introns. The diagram is drawn to scale. The accession numbers for OsCNGC genes are listed in Figure 1. (PNG 108 KB) [file 12864_2014_6538_MOESM1_ESM.png]

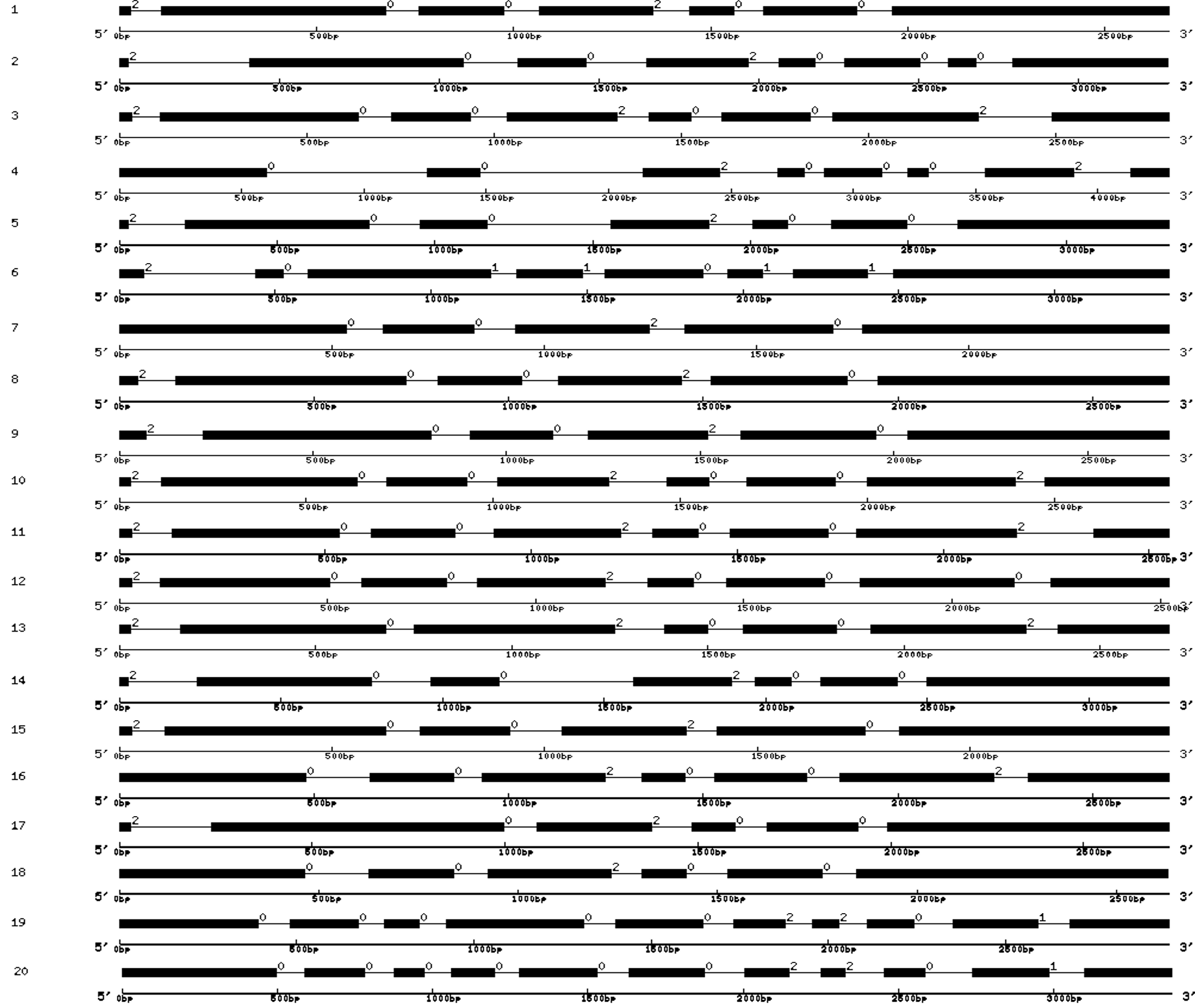

Supplement: Supplementary file 2 — Additional file 2: Schematic diagram representing structures of CNGC genes of Arabidopsis. Exons and introns are indicated as black boxes and black lines, respectively. Intron phase numbers 0, 1 and 2 are also shown at the beginning of the introns. The diagram is drawn to scale. The accession numbers for AtCNGC genes are listed in Additional file 9. (TIFF 742 KB) [file 12864_2014_6538_MOESM2_ESM.tiff]

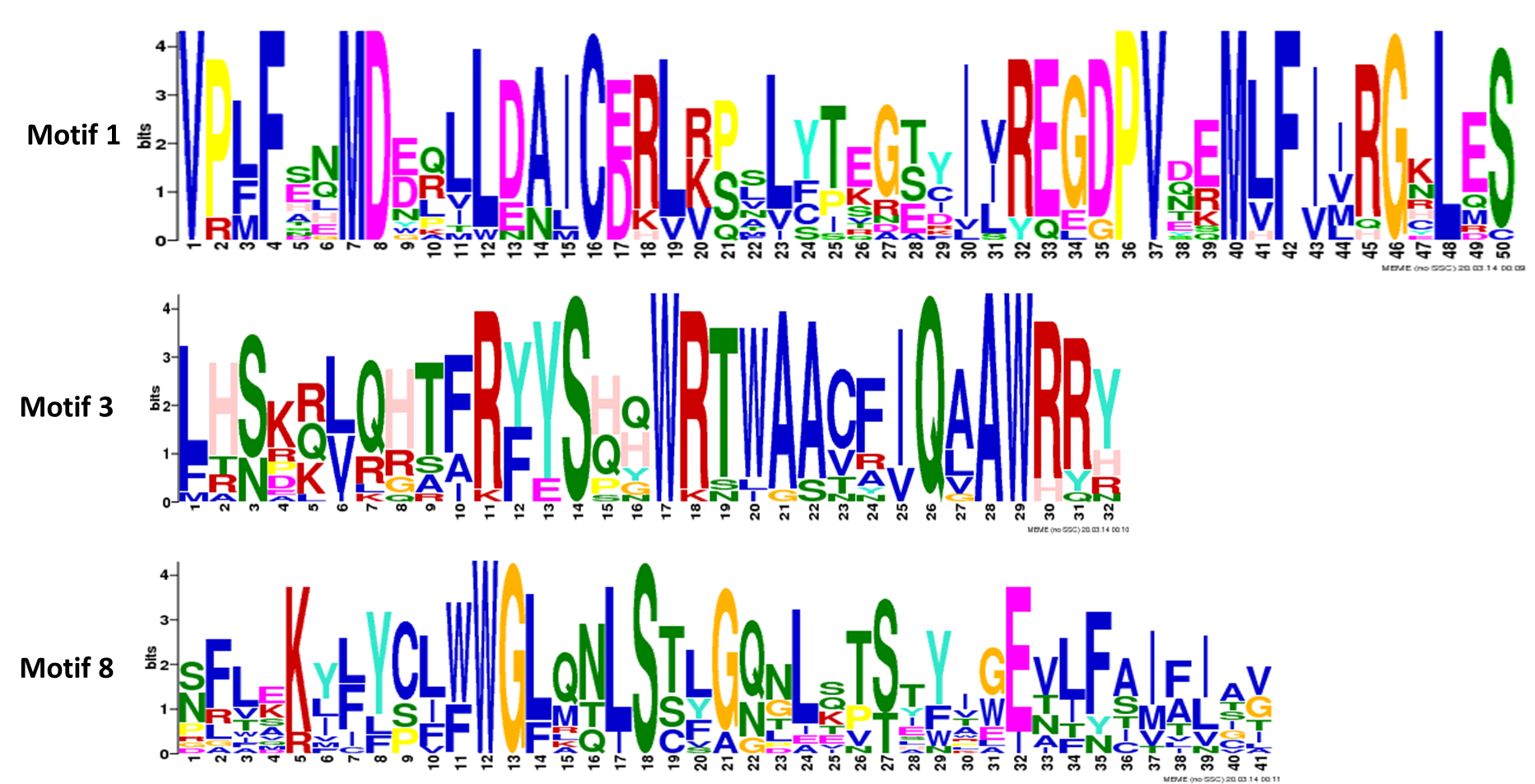

Supplement: Supplementary file 3 — Additional file 3: Sequence LOGOs for each motif of CNGC domains using the MEME algorithm. Motif 1: CNBD; Motif 3: IQ Motif 8: Ion transport. MEME motifs are displayed by stacks of letters at each position. The total height of the stack is the “information content” of that position in the motif in bits. The height of the individual letters in a stack is the probability of the letter at that position multiplied by the total information content of the stack. X- and Y-axis represents the width of motif and the bits of each letters, respectively. The details of motifs are given in Additional file 4. (PNG 621 KB) [file 12864_2014_6538_MOESM3_ESM.png]

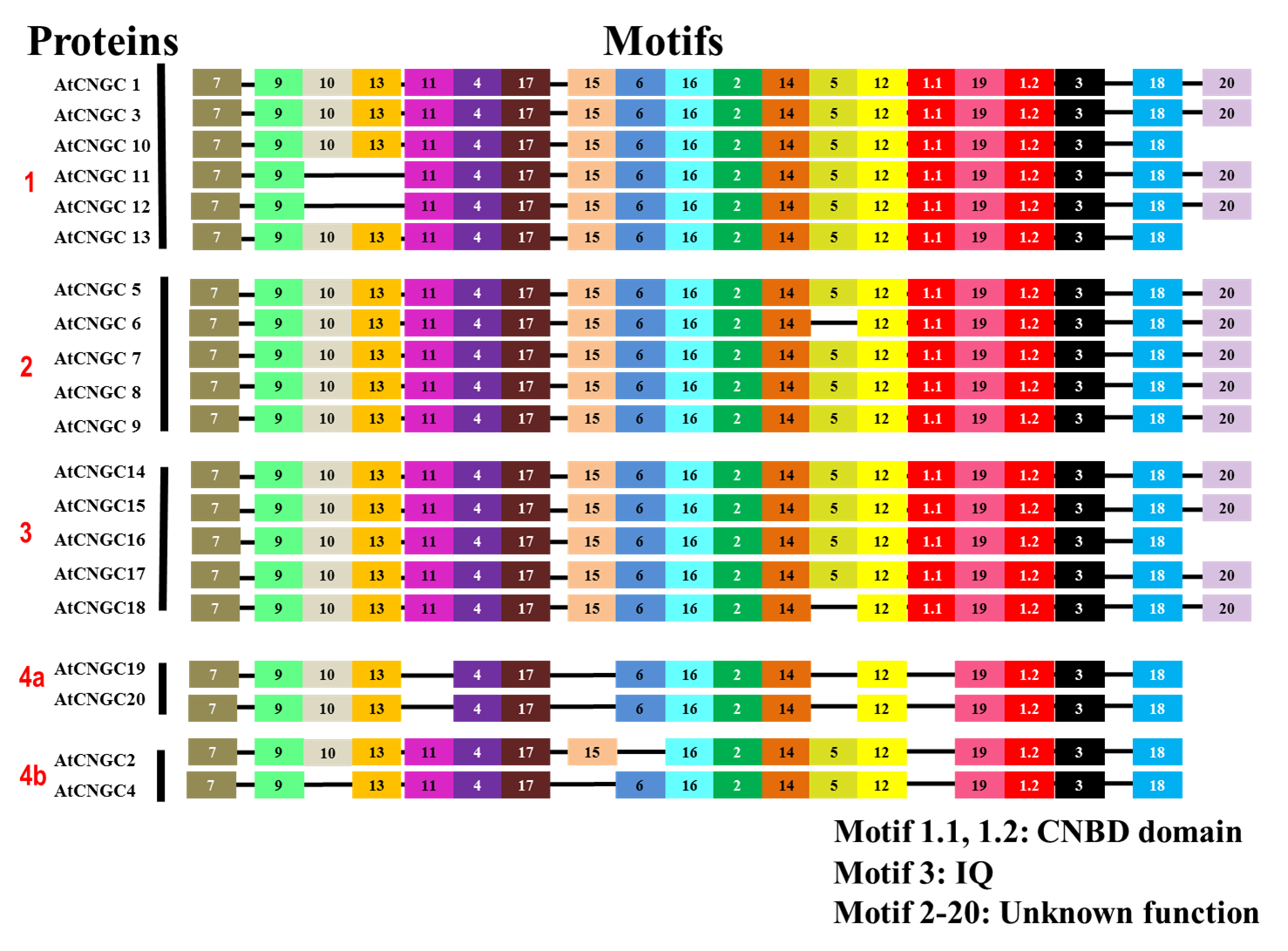

Supplement: Supplementary file 5 — Additional file 5: Distribution of Conserved motifs in Arabidopsis CNGC proteins identified using MEME search tool. Schematic representation of motif composition in AtCNGC proteins sequences using MEME motif search tool for each groups given separately. Each motif is represented by a number in colored box. Length of box does not correspond to length of motif. Order of the motifs corresponds to position of motifs in individual protein sequence. (TIFF 649 KB) [file 12864_2014_6538_MOESM5_ESM.tiff]

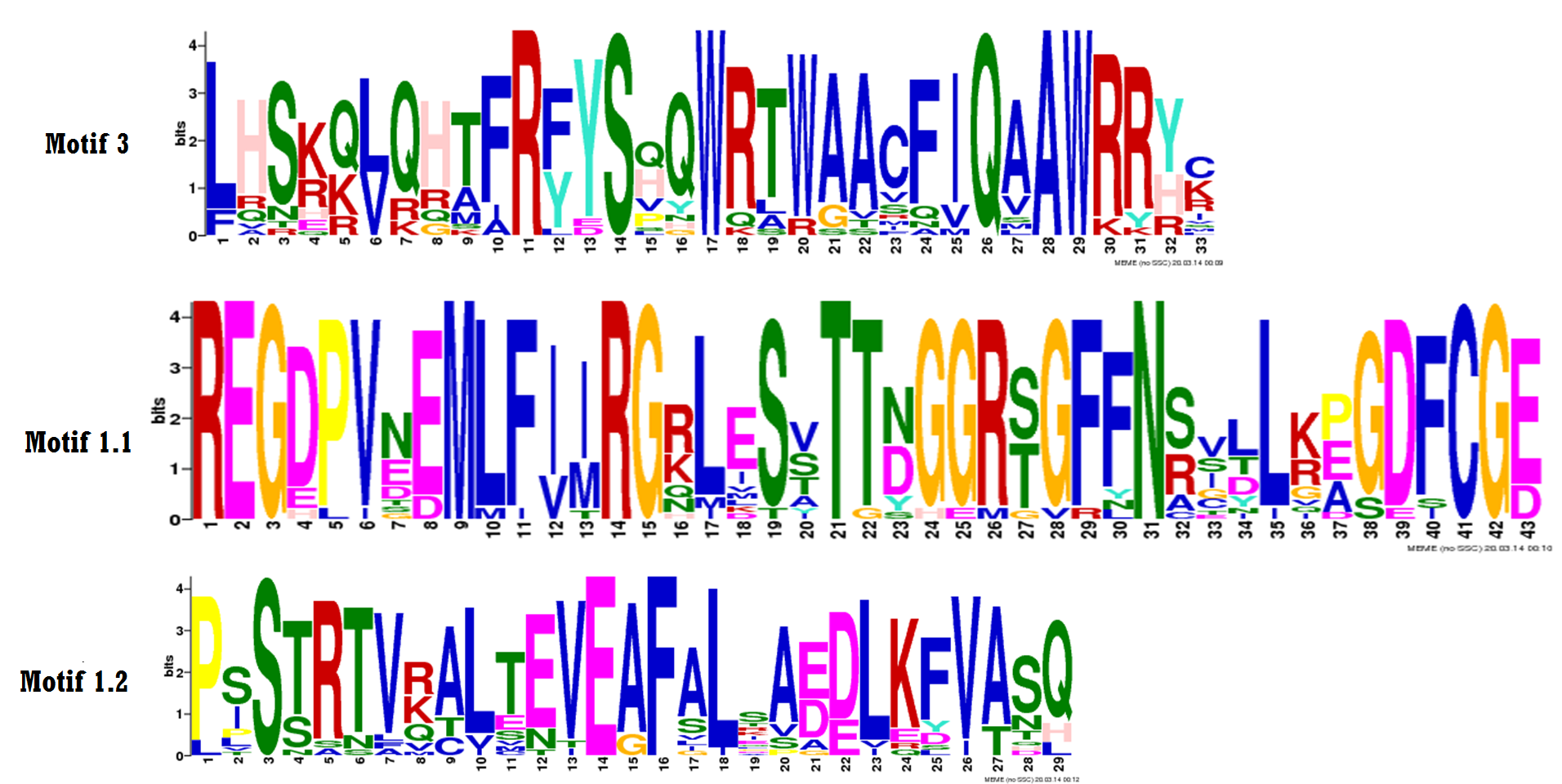

Supplement: Supplementary file 6 — Additional file 6: Sequence LOGOs for each motif of Arabidopsis CNGC domains using the MEME algorithm. Motif 2: IQ; Motif 3 & 8: CNBD. MEME motifs are displayed by stacks of letters at each position. The total height of the stack is the “information content” of that position in the motif in bits. The height of the individual letters in a stack is the probability of the letter at that position multiplied by the total information content of the stack. X- and Y-axis represents the width of motif and the bits of each letters, respectively. (PNG 597 KB) [file 12864_2014_6538_MOESM6_ESM.png]

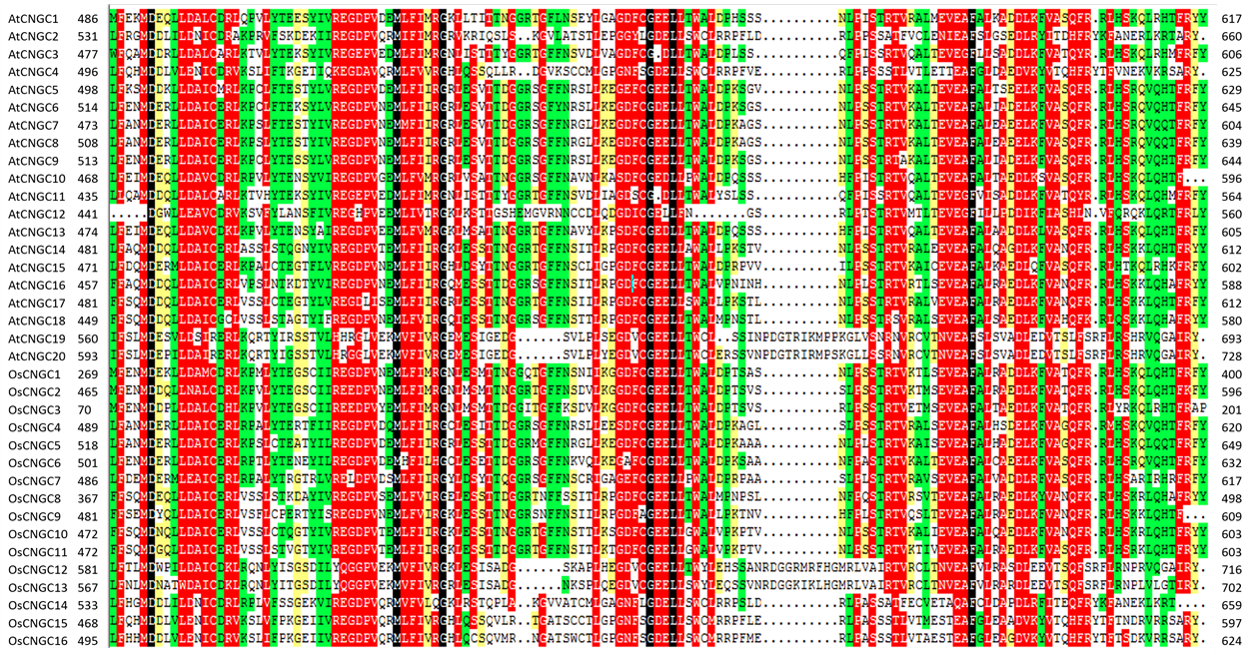

Supplement: Supplementary file 7 — Additional file 7: Multiple alignment profile of the CNBD domains of rice and Arabidopsis CNGCs obtained with ClustalX program. All the sequences show high level of amino acids conservation. Gaps (dashes) have been introduced to maximize the alignments. The most conserved feature of CNBD domain, the PBC and the hinge region are shown. The numbers at each end of the sequence show the start and stop positions of the region obtained from full length 36 CNGC protein. Followed by the names are the Residues highlighted in black indicate >100% conservation among the 36 CNGCs. Red highlighted residues indicates >70% identity. (PNG 1 MB) [file 12864_2014_6538_MOESM7_ESM.png]

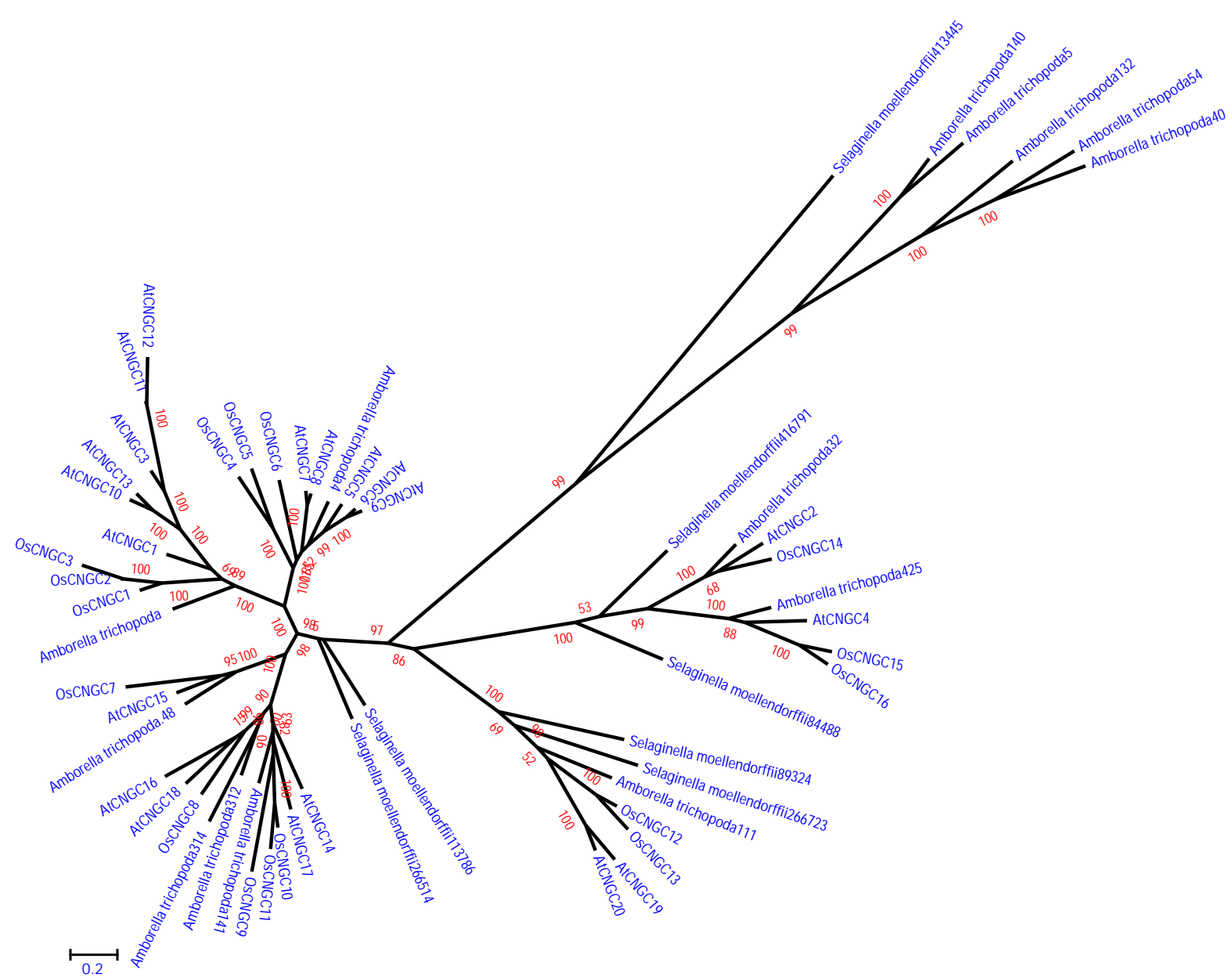

Supplement: Supplementary file 8 — Additional file 8: Phylogenetic tree of CNGC genes belonging to rice and Arabidopsis. A multiple sequence alignment was performed by MAFFT v7.017 with the L-INS-i model. The alignment was further used to construct a maximum likelihood phylogenetic tree by FastTree keeping the default settings. (PDF 11 KB) [file 12864_2014_6538_MOESM8_ESM.pdf]
