# Supplementary material for: Cyclic nucleotide-gated ion channel gene family in rice, identification, characterization and experimental analysis of expression response to plant hormones, biotic and abiotic stresses
Source: BMC Genomics. 2014 Oct 4;15(1):853. doi: 10.1186/1471-2164-15-853 (PMC4197254; doi:10.1186/1471-2164-15-853)
Supplement: Supplementary file 12 — Additional file 12: Expression profiles of OsCNGC genes in 4 weeks old rice leaves with UBQ5 internal control. Gene expression was perforemd by qPCR, 4 h after each treatment and non-treated CK. (A) Expression in response to different hormonal treatments. (B) Expression in response to pathogens inoculation with Xoo and P. fuscovaginae (biotic stress). (C) Expression in response to cold (abiotic stress). (DOCX 408 KB) [file 12864_2014_6538_MOESM12_ESM.docx]

Relative expression


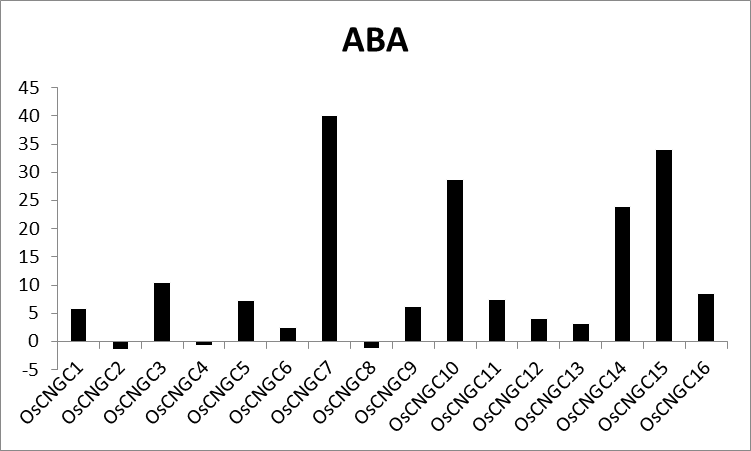

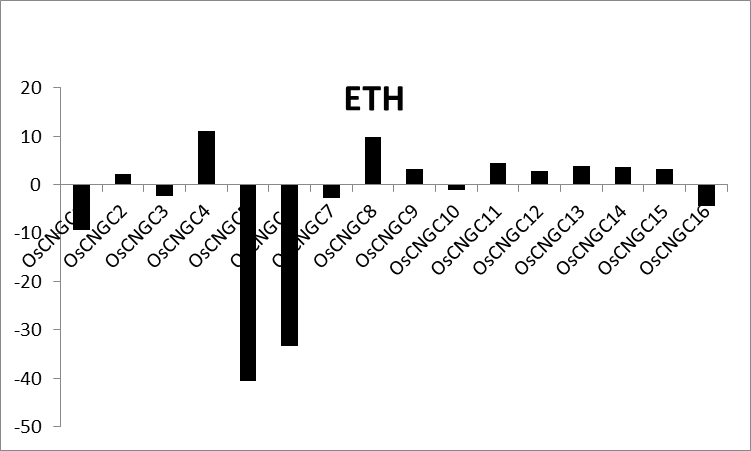

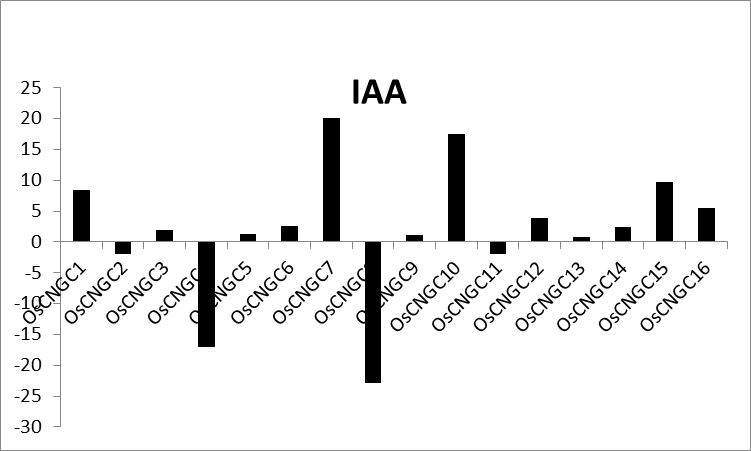

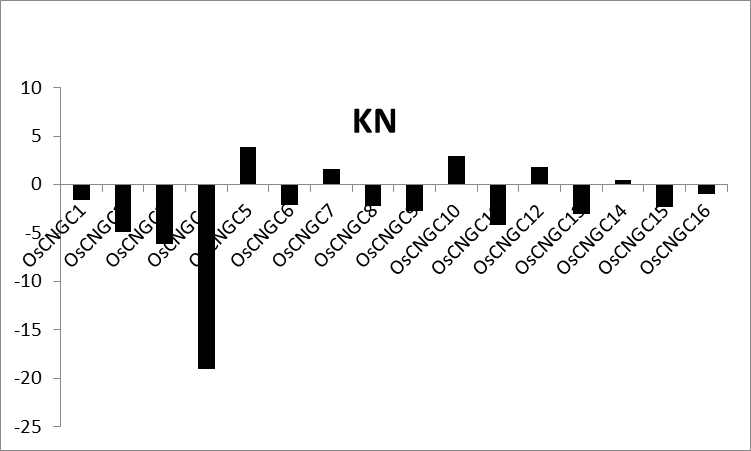

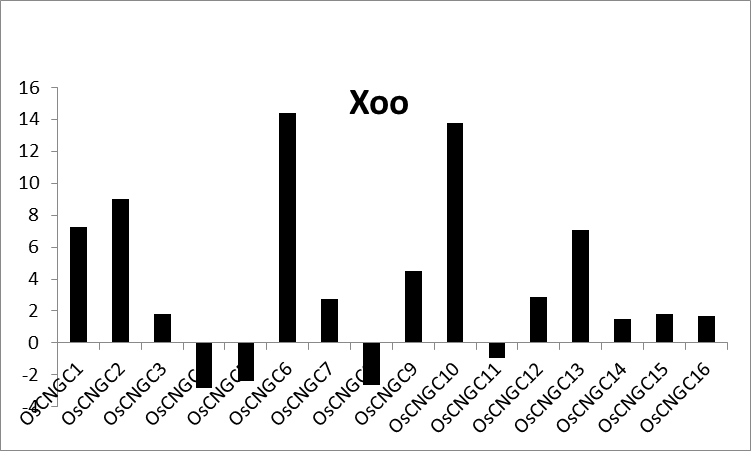

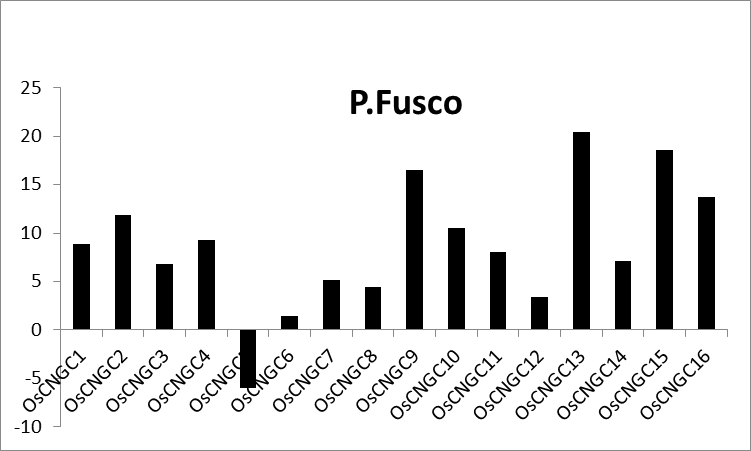

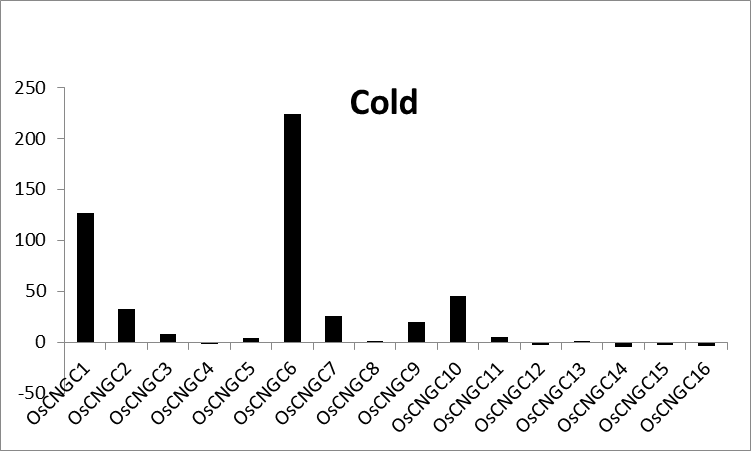


**A**

Relative expression

**A**

Relative expression

Relative expression

Relative expression

**C**

**B**

Relative expression

Relative expression
